# Transcriptomic, mutational and structural bioinformatics approaches to explore the therapeutic role of FAP in predominant cancer types

**DOI:** 10.1007/s12672-024-01531-x

**Published:** 2024-11-23

**Authors:** Gayathri Ashok, Abdullah F. AlAsmari, Fawaz AlAsmari, Paul Livingstone, Anand Anbarasu, Sudha Ramaiah

**Affiliations:** 1 Medical and Biological Computing Laboratory, School of Biosciences and Technology (SBST), Vellore Institute of Technology (VIT), Vellore, 632014 Tamil Nadu India; 2 Department of Bio-Sciences, SBST, VIT, Vellore, 632014 Tamil Nadu India; 3 Department of Pharmacology and Toxicology, College of Pharmacy, King Saud University, 11451 13 Riyadh, Saudi Arabia; 4 School of Sports and Health Sciences, Cardiff Metropolitan University, Cardiff, CF5 2YB UK; 5 Department of Biotechnology, SBST, VIT, Vellore, 632014 Tamil Nadu India

**Keywords:** Cancer-associated fibroblasts, Overall survival, Molecular docking, Molecular dynamics simulations, Mutations, Tumor microenvironment

## Abstract

Fibroblast activating protein (FAP) is a cell surface marker of cancer-associated fibroblasts with a distinct pro-tumorigenic role. The present study analyzed the pan-cancer expression; and clinical and mutational profiles of the FAP coding gene. Molecular dynamics simulation (MDS) deciphered the backbone dynamics and energetics of FAP. Virtual screening and subsequent pharmacokinetic-profiling (PK) filtered lead molecules, which were subjected to molecular docking. MDS projected a stable trajectory for the protein, as dynamics evidenced by low residue-level fluctuations, stable backbone dynamics, and energetics. Around five stabilization and deleterious mutations in the catalytic domain were identified. The low binding energy (BE) profiles from molecular docking studies screened the top five lead molecules for site-specific intermolecular interaction studies. Lead-16 (ZINC000245289699) exhibited a significant BE and inhibition constant of −6.87 kcal/mol and 12.27 μM, respectively, across FAP and its mutants. Interestingly, the docked complexes of Lead-16 interacted with the catalytic triad residues (S624, D702, and H734). The docked complexes of Lead-16 with FAP showed lower average root-mean-square fluctuations compared to the unbound protein, suggesting a stable ligand–protein complex. The tumor-specific expression and its critical overall survival suggest the inhibitors of FAP for potential cancer therapeutic intervention and hindering tumor microenvironment-driven cancer progression.

**Supplementary Information:**

The online version contains supplementary material available at 10.1007/s12672-024-01531-x.

## Introduction

Consistent increase in cancer cases and associated deaths in times of advancing therapeutics translates the adaptive measures acquired by the tumor cells for progressive tumorigenesis. Various facets and emerging hallmarks support the tumor to bypass the immune responses and drug effects thereby, leading to a more aggressive cancer phenotype that requires a targeted approach for circumvention [1]. One of the factors that supports aggressive tumorigenesis is the crosstalk between the tumor cells and surrounding microenvironment components. The tumor microenvironment (TME) plays diverse roles in driving tumorigenesis, such as inducing an immune suppressive environment, chronic inflammation, and promoting angiogenesis among others [2]. Crucial anti-tumor therapy is to target angiogenesis; however, most patients stop responding to the therapy due to the influence of TME. This can be due to the production of pro-metastatic proteins, initiation of tumor epithelial-mesenchymal transition or activation of compensatory pathways. One of the major components of TME is the cancer-associated fibroblasts (CAFs) formed by the oncogenic transformation of normal fibroblasts. The CAFs express fibroblast activating protein (FAP), a membrane-bound serine protease that is usually not detected in normal fibroblasts [3]. A few scenarios that reflected the potential role of FAP in promoting aggressive tumors were reported for breast, colorectal, pancreatic and gastric cancer. The increased FAP expression promoted ductal carcinoma that would not be otherwise promoted in the absence of FAP in breast cancer [4]. Similarly, in colorectal cancer (CRC) where FAP was detected in the cancer cells and surrounding stromal cells containing fibroblasts, an elevated expression of FAP were identified to be aiding in the development of CRC [5]. Interestingly, increased FAP expression in pancreatic cancer has been associated with lymph node metastasis and tumor recurrence [6].

FAP is a post-prolyl peptidase actively found on the cell surface, which has a dual role of endopeptidase and dipeptidyl peptidase activity. The protein also shares a close similarity (~ 40% homology) with dipeptidyl peptidase (DPP) family and differentiates it from them in the endopeptidase activity. The FAP acts on matrix metalloproteinases (MMPs) degraded collagens and alpha-2-antiplasmin. However, the interaction between the FAP and collagens depends on the prior degradation of collagen by MMPs or heat. FAP has been identified to play a role in cancer by modifying the bioactive signaling peptides through their enzymatic activity. It also undergoes dimerization with dipeptidyl peptidase IV (DPPIV) forming a heterodimer, and supporting the invadopodia of migrating fibroblasts [7]. The FAP in TME provides an immunosuppressive environment by promoting myeloid-derived suppressor cells (MDSC) indicating a pro-tumorigenic role of FAP. Additionally, cleaving neuropeptide Y by FAP leads to increased microvessel density, highlighting its pro-angiogenic functions in tumors. Moreover, activated PI3K signaling by the inactive FAP (FAP_S624A) was also reported [8]. The cancer-specific expression of FAP encourages the use of FAP as a potent target for cancer therapy. FAP-targeted radiotracers are extensively used for tumor imaging and FAP inhibitors such as LAF-237, PT-630 and talabostat are currently used. However, poor pharmacokinetic (PK) properties, such as poor bioavailability, high toxicity, lower self-life and high synthetic accessibility of the inhibitors press for alternate inhibitors [9, 10]. Therefore, alternative inhibitors can be a potential therapeutic solution for the inhibition of FAP. For the present study, we focused on identifying therapeutic potential of FAP. The tumor-promoting role of FAP was reported earlier; however, there were no published reports on the pan-cancer expression and mutational profiling of FAP to the best of our knowledge. We explored and validated the therapeutic potential of FAP across pan-cancer by investigating the following facets (a) Pan-cancer expression profile of FAP (b) Functional significance and survival outcomes of FAP (c) Stability and mutational profile of FAP protein (d) Identification of alternative lead molecules for FAP inhibition (e) Binding affinity and stability assessment of the protein-lead molecule complex. We designed the present study to provide a comprehensive view of FAP in tumorigenesis and also to identify potent lead molecules that have good binding affinity with FAP even in its mutant state. The FAP gene expression across various cancers and its functional and survival outcomes were estimated using computational approaches [11, 12]. Thereafter, backbone dynamics, molecular dynamics simulations (MDS) and mutational profiling across tumors filtered the stabilizing frequent mutations in FAP. Ligand-based virtual screening followed by PK profiling led to the screening of lead molecules, which were then studied for their intermolecular interaction profile. The candidate molecule with a high average binding affinity (BA) and stable ligand–protein complex is selected among all the protein targets. Our study proposes the use of biologically safe and effective lead molecule for inhibiting FAP across various tumors. Further, in vitro*-*in vivo experiments on the lead molecule can be useful in proposing the drug candidate as an anti-cancer therapeutic intervention.

## Materials and methods

### Pan-cancer expression profiling of FAP gene

The pan-cancer gene and protein expression profiling of FAP were conducted using the OncoDB server, which deals with large-scale multi-omics data retrieved from the public repository namely The Cancer Genome Atlas (TCGA). Currently, the database integrates the abnormal expression pattern across various clinical grades along with the methylation profiling. For the present study, we analyzed the expression profile of FAP across 34 cancers. The differential expression pattern was observed between the cancer sample median and normal sample median and the log2 fold change (FC) was retrieved at a significant p-value of < 0.05 [13].

### Retrieval of co-expression genes of *FAP*

Gene Expression Profiling Interactive Analysis (GEPIA) server was utilized for the filtering of co-expressed genes of FAP across tumors. GEPIA is a public semi-autonomous server comprising RNA-Sequencing and whole genome sequencing data of cancer patients deposited in TCGA and GTEx. The repository includes differential gene expression, survival and co-expressed genes in various cancers. The pair-wise gene co-expression analysis of the FAP gene is performed using Pearson correlation across TCGA Tumor and Pearson correlation coefficient (PCC) > 0.5 is considered for the present study [14].

### Functional enrichment analysis

Gene set enrichment analysis was performed using Shiny GO v0.77 server to filter out the enriched biological pathways and gene ontology terms (GO) such as biological process (BP), molecular functions (MF) and cellular components (CC). The server employs R packages and shiny framework to statistically analyze pathways and protein–protein interactions. For the present study, FAP and its co-expressed genes were subjected to pathway enrichment analysis and the pathways were filtered out based on fold enrichment (FE), false discovery rate (FDR) and genes involved in the pathways [15].

### Pan-cancer survival analysis

Overall survival analysis estimated using Kaplan–Meier Plotter gives the hazard ratio (HR), which is the probability of an event (death in case of cancer) to occur. The statistical analysis was performed with a significance of 95% confidence between two groups, namely, high expression and low expression. It estimates the median overall survival between the groups, thereby highlighting the impact of the gene expression on the overall survival [16, 17].

### Protein structure retrieval and refinement

The 3D structure of the FAP was retrieved from AlphaFold, which was then analyzed for Ramachandran favored regions [18]. Energy minimization of FAP was achieved through steepest descents and conjugate gradient algorithms for 2000 steps each using SPDBV with GROMOS96 43B1 forcefield *in vacuo* [19]. Secondary structure analysis was performed using SOPMA and PSIPRED servers to decipher the structural descriptors that highlight their functionality [20, 21], while the ProSA-web server was used to perform tertiary structure analysis. ProSA-web server computes a Z value corresponding to the overall quality score for the specific structure. The score is computed by taking into consideration the C-α atoms and is estimated by correlating with a group of structures from different sources such as X-ray diffraction or NMR spectrometry [22]. The instability index and hydropathicity of the protein were computed using the ProtParam tool [23]. The instability index estimates the stability of the protein of interest. Proteins with an instability value lesser than 40 are predicted to be stable, while proteins whose instability index values beyond 40 are considered unstable. The hydropathicity of a protein is calculated by the grand average of hydropathy divided by the number of residues in the protein sequence. The aliphatic index provides the relative volume engrossed by the aliphatic amino acids and is considered a positive indicator for the increasing thermostability for globular proteins. The characterized catalytic domain of the protein was then identified using the InterPro database [24].

### Stability assessment of protein through MDS

MDS was then performed to evaluate the stability of the target protein in an aqueous environment using the GROMACS 2020.2 package. The protein simulation was initiated by building topology and coordinates using CHARMM36 2020 forcefield. Thereafter, the protein was centered in the dodecahedron system by placing it at 1.2 nm from the edge and solvation was performed using a three-point solvent (TIP3P) model. The solvated system was then charge neutralized by adding ions (Cl^−^/Na^+^) using gmx genion by replacing the solvent molecule with the ions. Energy minimization of the system was then performed a maximum step size of 0.01 nm for 50,000 steps using the steepest descents method for removing any steric clashes. The system was then equilibrated in two phases, namely, NVT (constant Number of particles, Volume and Temperature) and NPT (constant Number of particles, Pressure and Temperature) ensembles for 100 ps, resulting in a canonical and isothermal-isobaric system. The resulting equilibrated system devoid of any position restraints was finally subjected to MD run for a timescale of 100 ns to obtain the MDS trajectory data [25, 26].

### Mutational profiling

The overall mutational rate of FAP was analyzed using the cBioPortal for genomics [27]. The portal integrates clinical and mutational data from different studies conducted across diverse cancers. Further, single nucleotide polymorphisms (SNPs) of FAP were identified using the BioMuta server. The web-based server categorizes the SNPs observed in different cancers and provides the frequency, amino acid change, and probable functional impact of the SNPs [28].

### Stability and functional impact of mutants

The functional impact of the SNPs was then analyzed using the PredictSNP server, which is a SNP analysis tool amalgamated with other prediction tools such as MAPP, nsSNPAnalyzer, PANTHER, PolyPhen-1, SIFT and SNAP. The server is improvised from the aforementioned servers in terms of unbiased performance due to overlapping datasets. The server computes the effect of mutations on the protein function (as neutral or deleterious) [29]. Thereafter, the impacts of the mutations on the protein structure were studied using the DynaMut server. The server predicts the structural impact based on the vibrational entropy (ΔΔS) and folding free energy (ΔΔG) [30].

### Structural assessment of the parent and mutant protein structures 

The SNPs corresponding to the catalytic sites were filtered and the mutant structures were generated by the standalone SPDBV software. The mutant protein and the wild protein structures were then subjected to structural assessment to understand the backbone dynamics through N–H S^2^ bond-order parameter using the Dynamine server. The server computes the residue level potential of a protein represented using S^2^ values from NMR chemical shift data. The value ranges from 0 to 1, depicting an increase in the rigidness [31].

### Ligand-based virtual screening and PK profiling

The existing small molecule inhibitors of FAP were determined from previous literature. Further, we employed the Search tool for Interacting Chemicals (STITCH) database, a public repository for interactions between proteins and chemicals. Using a medium confidence score (> 0.4), the chemical-target interactions were elucidated from different active interaction sources such as experiments, co-expression, predictions, textmining, co-occurrence, databases and gene fusion [32]. Thereafter, an FAP inhibitor (LAF-237) with better a PK-PD profile was taken as reference for the ligand-based virtual screening. Commercial lead-like molecules from the ZINC database were screened using the combined (2D and 3D) methods using the Swiss Similarity 2021 Web Tool. The screened molecules were provided with similarity scores ranging from 0 to 1 indicating the degree of similarity with increasing similarity score [33]. The ligands were then filtered by their PK profile performed using the SwissADME [34] and pkCSM [35] servers. Molecules surpassing GI absorption, and drug-likeness were then filtered using the Protox II server to predict the toxicity profiles including cytotoxicity, hepatotoxicity, carcinogenicity, mutagenicity or immunotoxicity. The server computes the median lethal dose (LD_50_) in mg/kg weight and toxicity class for the compounds [36]. The three-dimensional structure of the filtered analogs was generated using the Openbabel server [37].

### Intermolecular interaction profiling through molecular docking analysis

The shortlisted leads were then selected to understand the intermolecular interactions with the drug targets. The molecular docking among the drug targets namely, FAP and its mutants were performed using Autodock4.0 and its embedded tools [38]. The target proteins were optimized prior to docking analysis by adding polar hydrogens, merging non-polar atoms and Kollman charge addition. The ligand molecules were optimized by adding the Gasteiger charges and merging non-polar hydrogens. The grid box (60 Å x 60 Å x 60 Å) was centered at crucial active sites recognized from previous literature and InterPro domain analysis. The active site was validated upon understanding the surface topology of the protein using CASTp server that detects probable drug binding pockets based on their surface area (Å^2^) and volume (Å^3^) [39]. Molecular docking was then performed using Lamarckian and Genetic algorithms to predict the least binding energy ligand-target conformations. The docked complexes (with least binding free energies) were then visualized using the Discovery Studio Visualizer 2020.

### Dynamic simulations

Dynamic simulations of the protein structures were performed using the CABS-Flex 2.0 web server. The server predicts the residue-level dynamicity in the form of root mean square fluctuation (RMSF) using Monte Carlo dynamics and asymmetric metropolis scheme explaining the dynamics of the protein. The coarse-grained simulation was performed with default parameters having a gap of 3 Å, minimum and maximum field of 3.8 Å and 8.0 Å respectively, for both SS2 restraints. The RMSF values for the unbound protein and docked complexes were analyzed to determine the average deviation of the protein residue from the reference position [40].

**Fig. 1:**
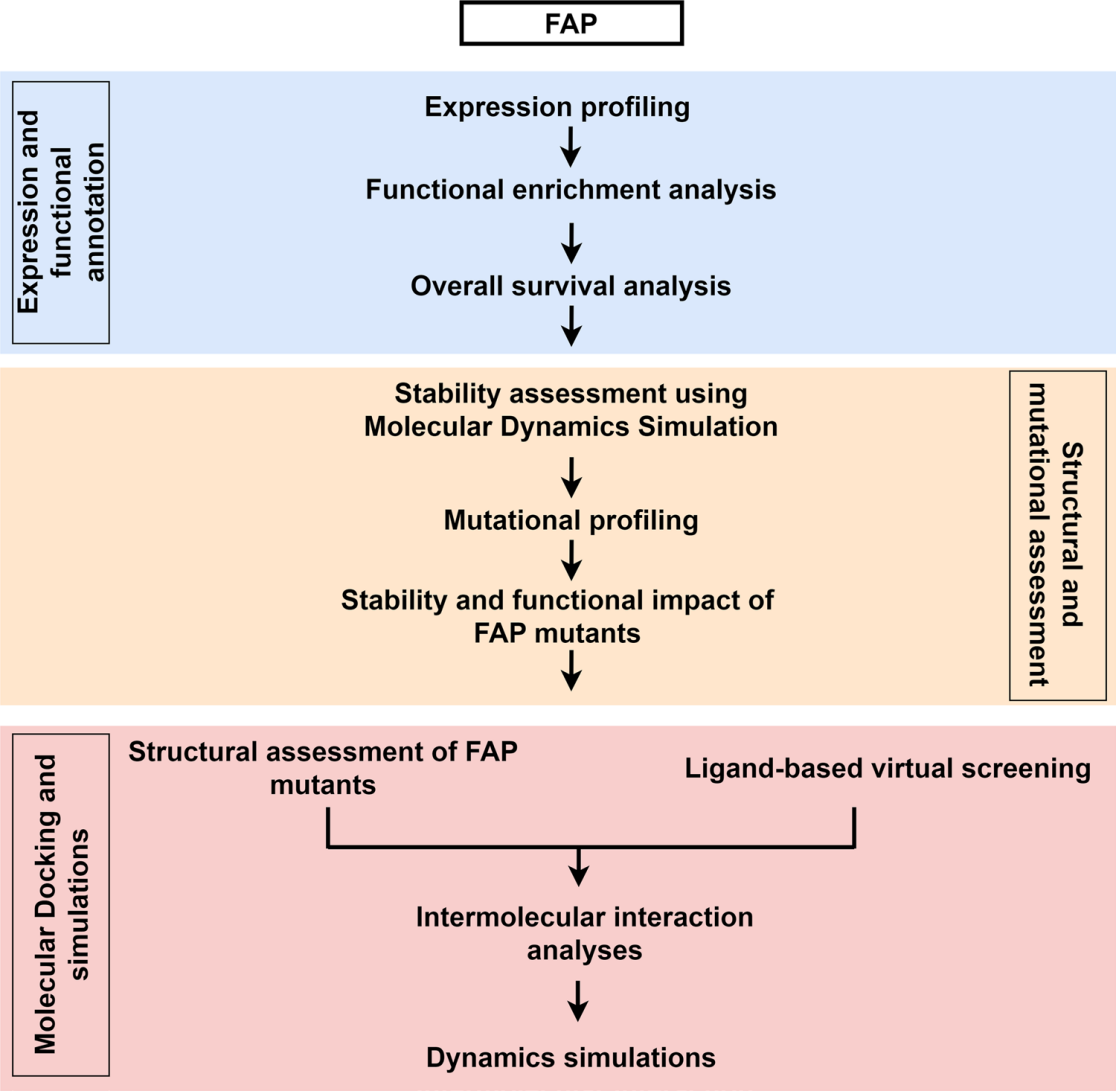
Schematic workflow depicting the overall analyses employed in the study

A comprehensive schematic workflow depicting the various analyses employed in the study design is represented in Fig. 1.

## Results

### FAP gene expression across cancers

**Fig. 2:**
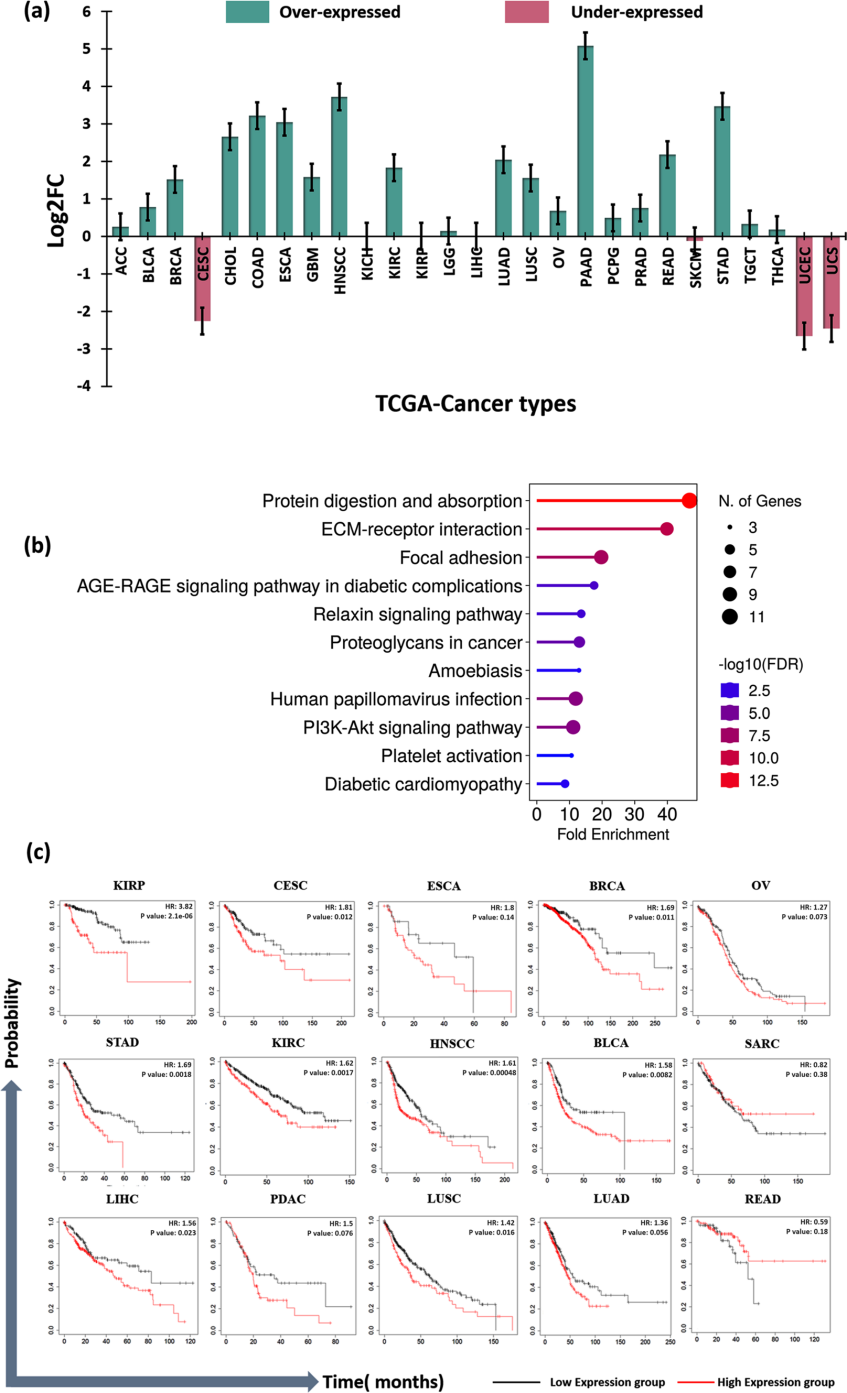
Clinical and Functional Enrichment of FAP **a** Expression pattern of FAP gene across various cancers types **b** Biological pathways enriched by FAP and its co-expressed genes **c** Kaplan–Meier Plot depicting overall survival of FAP gene across multiple cancer types

Pan-cancer expression analysis of FAP on 31 different types of cancer identified the increased expression of FAP in tumors compared to normal tissues. The gene expression (expressed in terms of log2FC) was evidently higher in Breast invasive carcinoma (BRCA) (1.52), Cholangio carcinoma (CHOL) (2.66), Colon adenocarcinoma (COAD) (3.22), Esophageal carcinoma (ESCA) (3.05), Glioblastoma multiforme (GBM) (1.58), Head and neck squamous cell carcinoma (HNSCC) (3.72), Kidney renal clear cell carcinoma (KIRC) (1.83), Lung adenocarcinoma (LUAD) (2.05), Lung squamous cell carcinoma (LUSC) (1.56), Pancreatic adenocarcinoma (PAAD) (5.08), Rectum adenocarcinoma (READ) (2.18) and Stomach adenocarcinoma (STAD) (3.47) as depicted in Fig. 2a (Online Resource 1).

### Functional enrichment analysis based on co-expressed genes

The co-expressed genes of FAP across cancers were then shortlisted with genes having a Pearson correlation coefficient > 0.5 (Online Resource 2). The major co-expressed genes were COL5A2 (PCC: 0.76), COL6A3 (PCC: 0.73), MMP2 (PCC: 0.72), COL1A2 (PCC: 0.72), CTHRC1 (PCC: 0.72), COL3A1 (PCC: 0.7), COL5A1 (PCC: 0.7) and ADAM12 (PCC: 0.7). Thereafter, FEA identified statistically significant enriched pathways to be protein digestion absorption (FE: 46.81), ECM-receptor interaction (FE: 39.85), focal adhesion (FE: 19.72), PI3K/AKT signaling pathways (FE: 11.14) and proteoglycans in cancer (FE: 13.02) (Fig. 2b).

### Overall survival analysis of FAP gene

The overall-survival analysis of the FAP gene was then compared across multiple cancer types to estimate the HR and median survival at a significant p-value. The cancers on which the high expression of FAP corresponded to high HR and low median survival were Bladder urothelial carcinoma (BLCA) (HR: 1.58), BRCA (HR: 1.69), Cervical squamous cell carcinoma (CESC) (HR: 1.81), ESCC (HR: 1.81), HNSC (HR: 1.61), KIRC (HR: 1.62), Kidney renal papillary cell carcinoma (KIRP) (HR: 3.82), Liver hepatocellular carcinoma (LIHC) (HR: 1.56), LUAD (HR: 1.36), Ovarian serous cystadenocarcinoma (OV) (HR: 1.27), Pancreatic ductal adenocarcinoma (PDAC) (HR: 1.5) and STAD (HR: 1.69) (Fig. 2c). Evident decrease in median survival of high expression groups were identified in BLCA, BRCA, KIRC, KIRP, LIHC, LUSC, PDAC and STAD (Online Resource 3).

### Protein structure refinement and structural assessment

**Fig. 3:**
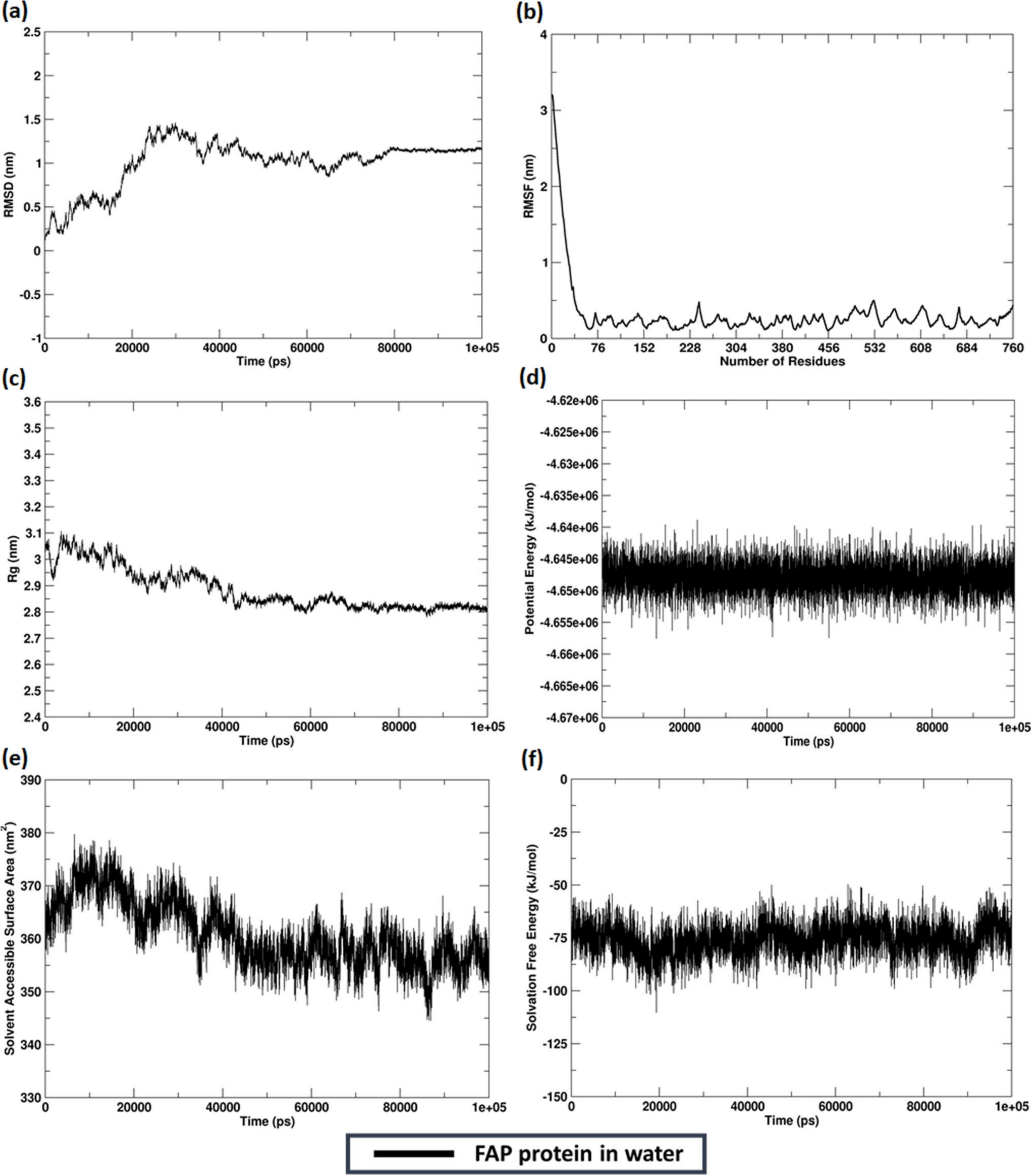
Molecular dynamics simulation studies of FAP **a** RMSD curve **b** RMSF- Residue level fluctuations **c** Radius of gyration plot **d** Potential energy curve **e** SASA plot **f** Solvation free energy plot

The 3D structure of FAP was retrieved from AlphaFold with the accession ID: AF-Q12884-F1. The protein with an initial energy (Ei: -33,917.06 kJ/mol) was then minimised (Ef: -44,694.71 kJ/mol) (Online Resource 4a). The secondary structure analysis identified FAP to be structurally well characterized with α-helix (20.26%), extended strand (30.13%), β-turns (6.18%) and random coil (43.42%) as depicted in Online Resource 4b. The overall model quality of the 3D structure analysed through tertiary structure analysis estimated a Z-value of -10.85 indicating an overall good model (Online Resource 4c-d). We computed the instability index of the protein, which was identified to be 38.7. The aliphatic index and hydropathicity of the protein were estimated to be 91.45 and -0.294 respectively. Thereafter, the catalytic domain of the protein was found to be peptidase S9/prolyl oligopeptidase domain (IPR001375) spanning across 555–757 amino acid residues (Online Resource 4e). MDS analysis translated the stability profile of the protein, indicating a root-mean-square-deviation (RMSD) of 1.006 ± 0.275 nm (Fig. 3a). While studying the average residue-level fluctuations of the protein, it was identified that the overall root-mean-square-fluctuation (RMSF) was 0.310 ± 0.376 nm, while the catalytic domain had an RMSF of 0.250 ± 0.07 nm (Fig. 3b). The stable tertiary protein structure was depicted by the constant value of the radius of gyration (R_g_) of 2.884 ± 0.078 nm, indicating the compactness of the structure (Fig. 3c). Further, analyzing the potential energy (P_E_) profile of the protein during simulation was found to be energetically favorable with a P_E_ of -4.67e + 06 kJ/mol (Fig. 3d). Thereafter, the folding and stability of the protein was evaluated using SASA area and free energy of solvation. The SASA of the protein was 360.938 ± 6.049 nm^2^ (Fig. 3e), while the free energy solvation of the protein was -75.836 ± 7.325 kJ/mol (Fig. 3f).

### Mutational profiling of FAP

**Fig. 4:**
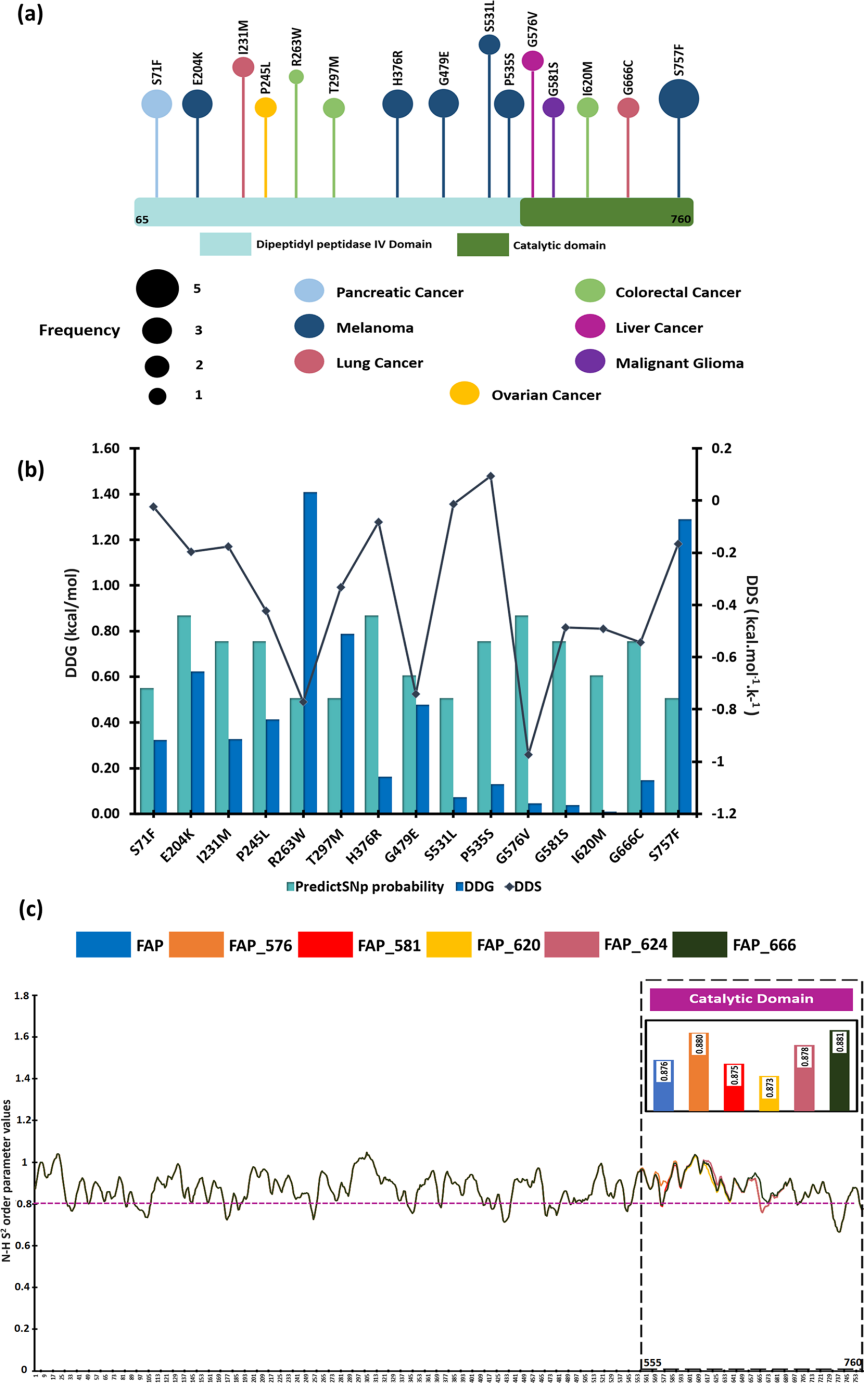
Mutational Profiling of FAP **a** The stabilizing and deleterious mutations identified in the catalytic domain across multiple cancer types **b** The stabilizing, decreasing flexibility and deleterious mutations filtered in the catalytic domain **c** Backbone stability profile of FAP and mutant proteins identified using N–H S^2^ parameter

The mutational profiling of the FAP showed a 2.5% mutation rate including missense mutations, amplification and deletion. The analysis was conducted across 13,867 samples (across two studies namely, MSK-IMPACT clinical sequencing with 10,945 samples and Pan-cancer analysis of whole genome with 2922 samples). Further, we listed 172 non-synonymous mutations (SNPs) in FAP across multiple tumors (Online Resource 5A). Thereafter, the functional and structural characterization of the SNPs (with frequency ≥ 2) identified 15 SNPs, which were functionally deleterious with increasing stability (ΔΔG) and decreasing flexibility (ΔΔS) as depicted in Fig. 4a-b. Among the filtered mutants, only five SNPs were characterized to be in the catalytic domain namely, G576V, G581S, 1620 M, G666C and S757F. The SNPs were found in liver, glioma, colorectal, lung and melanoma cancers (Online Resource 5B). Additionally, we also considered catalytically inactive mutation (S624A) for further analyses.

### Structural stability analysis

The 3D structure of the mutants was generated using SPDBV software and was then subjected to energy minimization. The deleterious mutation S757F cannot be induced in the structure due to steric hindrance. Thereafter, the structural assessment of the protein and the mutants was evaluated by backbone dynamics depicted through the N–H S^2^ bond-order parameter. The average N–H S^2^ for FAP was 0.873, while the mutants FAP_S576V and FAP_G666C had a N–H S^2^ value of 0.875, FAP_G581S and FAP_I620M had N–H S^2^ value of 0.873 and FAP_S624A had N–H S^2^ value of 0.874. While analyzing the average change in the backbone dynamics at the catalytic domain in the mutant proteins with respect to the wild protein (FAP), it was observed that FAP_S576V (0.4%), FAP_S624A (0.2%) and FAP_G666C (0.5%) showed a slight increase in the N–H S^2^ order while FAP_G581S (− 0.1%) and FAP_I620M (-0.3%) showed slight decrease in the N–H S^2^ order parameter (Fig. 4c).

### Ligand screening and ADMET property analysis

The conventionally used small molecule inhibitors for FAP were determined from previous literature (Talabostat, PT-630 and LAF-237) and STITCH database (CID046917048, CID071655266, CID066954220, CID044590651, CID071655265, CID044590650, CID067507176, CID067507912 and CID010096344) (Online Resource 6). LAF-237 was considered as a reference compound for FAP upon which virtual screening was performed to obtain commercial small lead-like molecules based on 2D and 3D parameters from the ZINC database. The 400 analogs screened shared a similarity score ≥ 0.977 with LAF-237. The ADMET profile of the screened compounds resulted in of 33 compounds with no Lipinski violations, high GI absorption, high bioavailability score, low synthetic accessibility, high LD_50_ and no reported toxicity as summarized in Table 1. Also, it was noted that all the reference compounds showed poor PK properties (low LD_50,_ low GI absorption, lead-like violations and comparatively high synthetic accessibility) when compared to the screened molecules.

**Table 1:** PK profile of screened lead molecules and reference compound

Lead molecules	LD_50_ (mg/kg)	GI absorption	Pg substrate	Lipinski violations	Bioavailability score	Leadlikeness violations	Synthetic accessibility	2D structures
Lead 16 (ZINC000245289699)	5000	High	No	No	0.55	No	3.4	
Lead 8 (ZINC000096419372)	5000	High	No	No	0.55	No	3.4	
Lead 21 (ZINC000248057745)	5000	High	No	No	0.55	No	3.24	
Lead 29 (ZINC000583650907)	3000	High	No	No	0.55	No	3.74	
Lead 17 (ZINC000245289700)	5000	High	No	No	0.55	No	3.4	
LAF-237 (CID: 6,918,537)	80	High	No	No	0.55	No	4.76	

### BA profile of docked complex

**Fig. 5:**
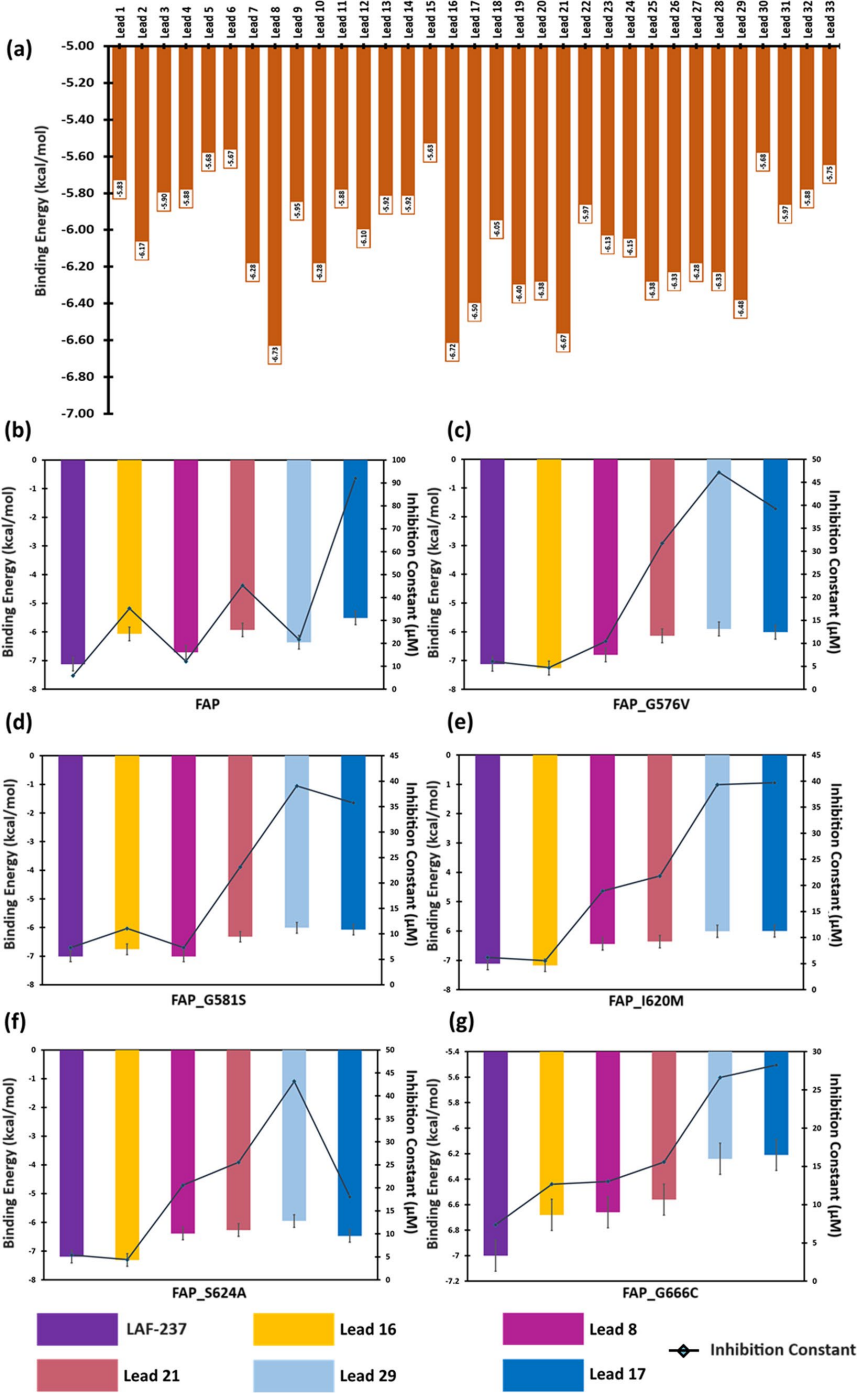
Binding energy profile **a** Virtual screening of ADME-T screened lead molecules **b** BE and IC of docked complexes of FAP and lead molecules **c** BE and IC of docked complexes of FAP_G576V protein and lead molecules **d** BE and IC of docked complexes of FAP_G581C protein and lead molecules **e** BE and IC of docked complexes of FAP_I620M protein and lead molecules **f** BE and IC of docked complexes of FAP_S624A protein and lead molecules **g** BE and IC of docked complexes of FAP_G666C protein and lead molecules

**Fig. 6:**
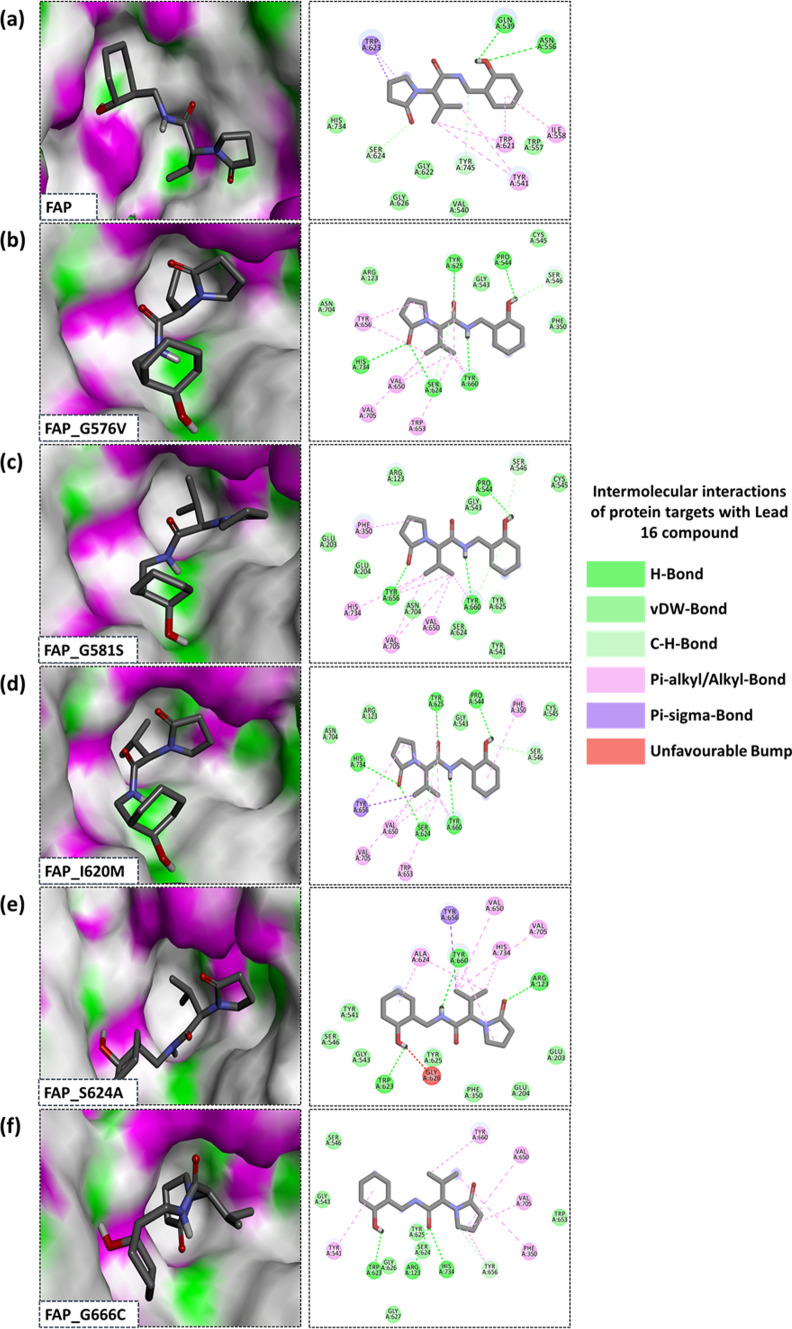
Intermolecular interactions of docked complexes of protein targets with Lead 16 **a** FAP **b** FAP_G576V **c** FAP_G581S **d** FAP_I620M **e** FAP_S624A **f** FAP_G666C

The binding affinity of the screened analogs with all six target proteins (FAP and mutants) was studied using AutoDock 1.5.7. Among the screened 33 compounds (which are termed as lead molecules from here onwards), virtual screening identified Lead 8, Lead 16, Lead 21, Lead 17 and Lead 29 to be filtered for site-specific intermolecular interactions, BA and IC profiling (Fig. 5a). Among the filtered five lead molecules, “Lead 16” showed high BA with all the protein targets with an average BE (BE_avg_) of -6.67 kcal/mol and average IC (IC_avg_) of 12.27 μM, followed by “Lead 8” with a BE_avg_ of -6.66 kcal/mol and IC_avg_ of 13.73 μM. However, LAF-237 showed a BE_avg_ of -7.09 kcal/mol and IC_avg_ of 6.37 μM as listed in Online Resource 7A. “Lead 16” showed a better binding affinity with mutants namely, FAP_S576V (-7.26 kcal/mol), FAP_I620M (-7.17 kcal/mol) and FAP_S624A (− 7.31 kcal/mol) than LAF-237 (Fig. 5b–g). The intermolecular interactions among the docked complex of “Lead 16” with FAP showed two hydrogen bonds, two carbon-hydrogen bonds and other non-canonical interactions such as van der Waals (vdW), Pi-alkyl, alkyl and Pi-sigma (Fig. 6a). Similarly, five H-bonds and two C-H bonds along with other non-canonical interactions with FAP_G576V (Fig. 6b). In case of FAP_G581S complexed with Lead 16 (Fig. 6c), the complex is stabilized by three H-bonds, two C-H bonds and non-covalent interactions such as vdW and Pi-alkyl and alkyl interactions. The high BA of FAP_I620M with Lead 16 can be attributed to five H-bonds, a single C-H bond and non-covalent interactions (Fig. 6d). Docked complex of FAP_S624A and Lead 16 showed three H-bonds and other non-covalent interactions as depicted in Fig. 6e. The Fig. 6f shows the docked complex of FAP_G666C and Lead 16 having three H-bonds, a single C-H bond along with other non-covalent interactions. The Lead 16 molecule was identified to be interacting with the residues of catalytic triad of FAP (vdW: H734; C-H bond: S624); FAP_G576V (H-bond: S624, H734; C-H bond: S624); FAP_G581S (vdW: S624; Pi-alkyl/alkyl: H734); FAP_I620M (H-bond: H734, S624); FAP_S624A (Pialkyl/alkyl: A624, H734) and FAP_G666C (H-bond: H734; vdW: S624) (Online Resource 7B). The 3D and 2D interaction of docked complexes of LAF-237, Lead 8, Lead 21, Lead 29 and Lead 17 is represented in Online Resource 8.

### Dynamic simulations of docked complexes

**Fig. 7:**
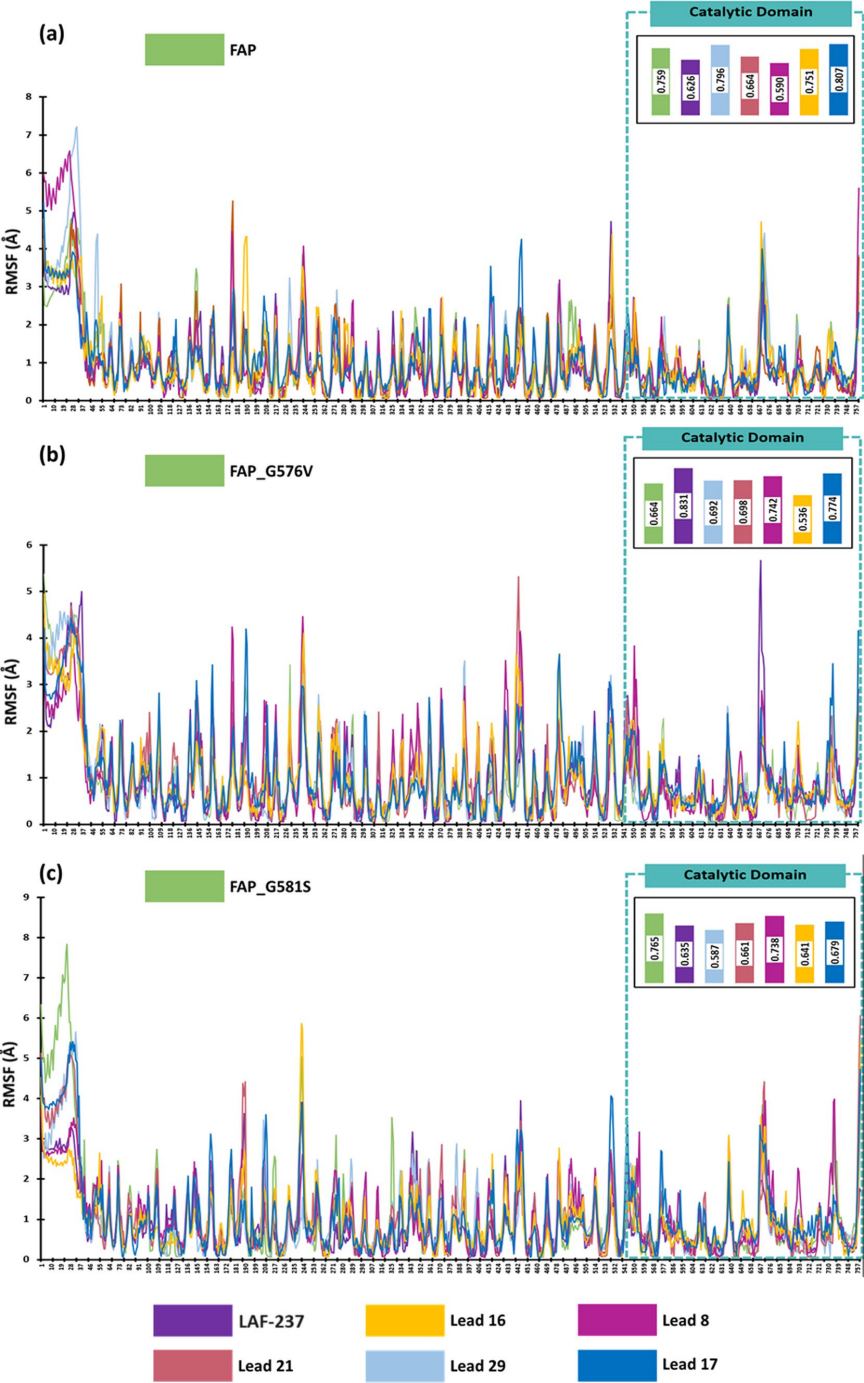
RMSF profile of the docked complexes **a** FAP **b** FAP_G576V **c** FAP_G581S

**Fig. 8:**
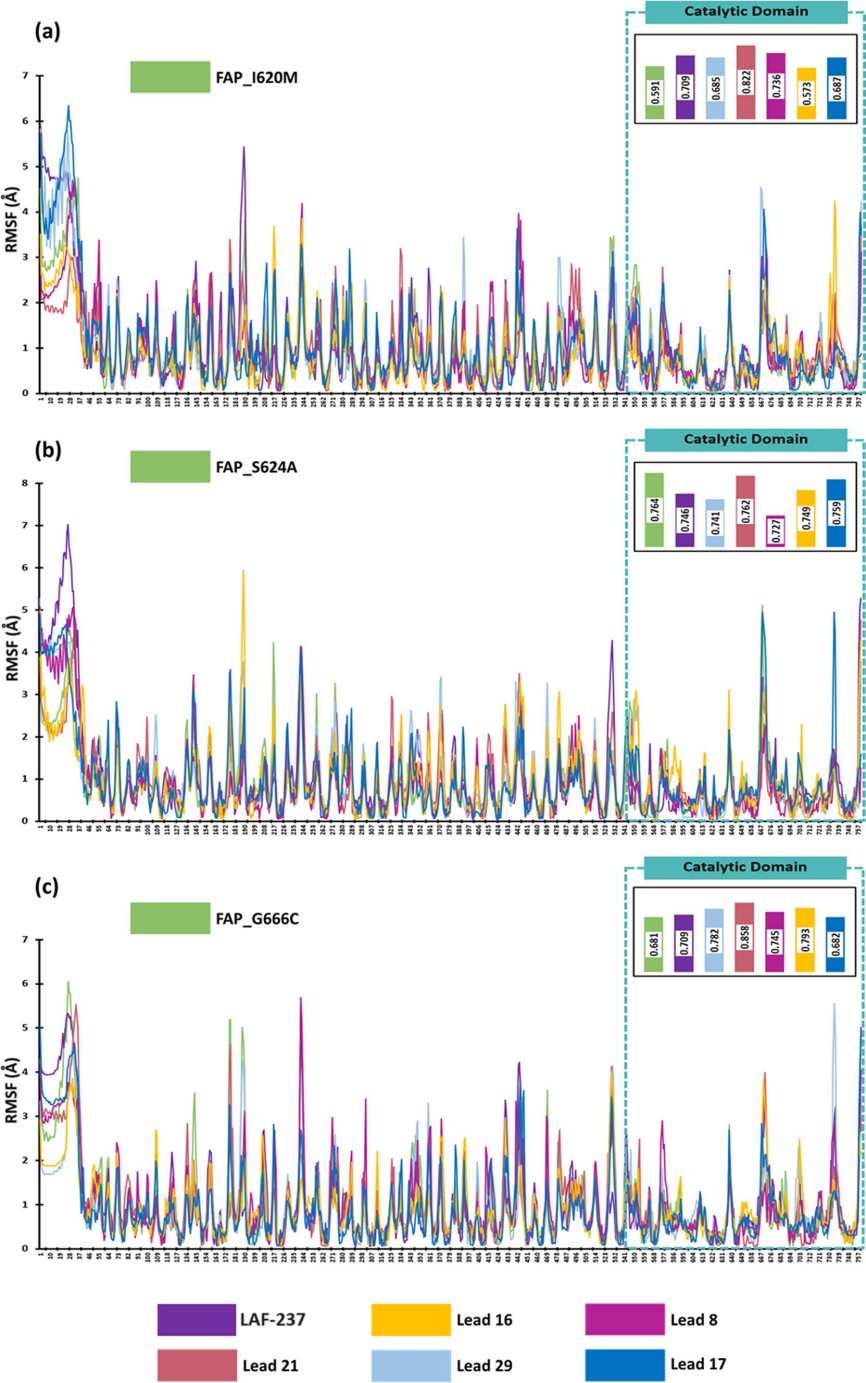
RMSF profile of the docked complexes **a** FAP_I620M **b** FAP_S624A **c** FAP_G666C

Dynamics simulation of the docked complexes evaluated the residual level fluctuations as compared to the unbound target proteins. When compared to the unbound FAP wild type protein (0.759 Å), docked complex of LAF-237 (0.626 Å), Lead 16 (0.751 Å), Lead 8 (0.590 Å) and Lead 21 (0.664 Å) showed lower average RMSF in the catalytic domain (Fig. 7a). In case of FAP_G576V protein, the average RMSF was identified to be 0.664 Å, while the docked complex with Lead 16 only depicted lower average RMSF of 0.536 Å, while all other lead molecules and LAF-237 showed evident higher average RMSF (Fig. 7b). For protein FAP_G581S (Fig. 7c), all lead molecules namely, Lead 16, Lead 8, Lead 21, Lead 29, Lead 17 and LAF-237 showed lower RMSF than unbound protein (0.765 Å) with an average RMSF of 0.641 Å, 0.738 Å, 0.661 Å, 0.587 Å, 0.679 Å and 0.635 Å respectively. Lead 16 depicted lower average RMSF when complexed with FAP_I620M showing an average RMSF of 0.573 Å when compared to unbound protein (0.591 Å), while the LAF-237 and lead compounds showed slightly higher average RMSF as depicted in Fig. 8a. From Fig. 8b, it is evident that docked complexes of lead molecules with FAP_S620M, showed lower average RMSF than the unbound protein which had an average RMSF of 0.764 Å. Unbound FAP_G666C protein showed an average RMSF of 0.681 Å, while only the docked complex of Lead 17 with FAP_G666C showed a lower average RMSF of 0.682 Å (Fig. 8c). Overall, Lead 16 formed less fluctuating docked complex with all the protein targets, suggesting a stable interaction profile with the protein targets.

## Discussion

Developing potent therapeutic interventions specific to cancer cells becomes imperative with the continuous adaptation of tumor cells to conventional therapies. TME is a critical factor that plays crucial roles in resisting tumor therapies by stimulating an immunosuppressive environment, promoting angiogenesis and other pro-tumorigenic factors [41]. The comprehensive evaluation of FAP across tumors identified it to be specifically expressed in tumor samples when compared to normal samples. Mutational profiling of the FAP identified stabilizing mutants such as FAP_G576V, FAP_G581S, FAP_I620M, and FAP_G666C in addition to catalytically inactive FAP_S624A. Ligand-based virtual screening and subsequent PK profiling filtered lead molecules that were further studied with the mutants to analyze the intermolecular interactions and stabilization profile.

The tumor-specific expression patterns evidenced in FAP were further supported by the differential expression observed between the tumor and normal tissues of various cancers. Poor prognosis has been associated with high intratumoral expression of FAP in colon cancer [42]. Similarly, elevated expression of FAP has also been associated with worse prognosis and promoting metastases in gastric cancer [43] and KIRC [44], respectively. This signifies the vital role of FAP in promoting tumors. The functional role of FAP and its co-expressing genes highlighted the tumor-promoting pathways such as focal adhesion, ECM receptor interaction, PI3K/Akt signaling and proteoglycans in cancer. The FAP-associated pathways are highly interlinked with the TME-related pathways for tumor progression. For instance, the proteoglycans in cancer are involved in the remodeling of the surrounding tumor by interacting with the growth factors and pro-tumorigenic molecules to aid in tumor progression [45]. The FAP overexpression leads to the promotion of migration, invasion and proliferation by up-regulating the PI3K and sonic hedgehog signaling [46, 47].

**Fig. 9:**
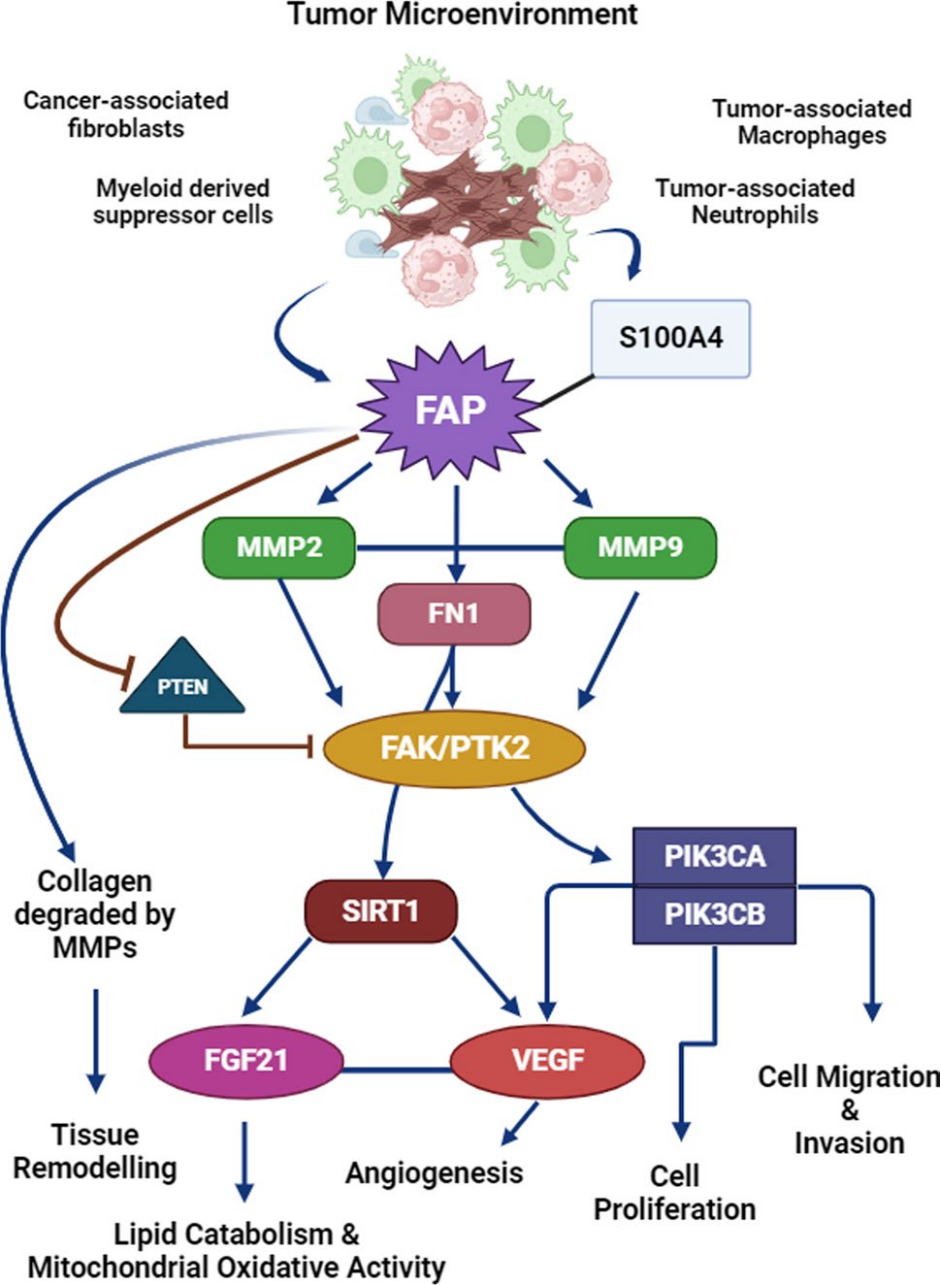
Schematic representation of the functional role of FAP in promoting tumorigenesis, migration and tissue remodelling

The FAP regulates the focal adhesion kinase (FAK) pathway by reducing the phosphorylation of FAK, leading to the increased number of focal adhesions, affecting the direction of migration in cancer cells. The highly regulated process of cell migration depends on the activation and disassembly of these adhesions formed by the activation of FAK. Continued formation of adhesions aids the tumor cells to metastasize, thereby promoting aggressive tumors inside the body [48–50]. This is in support of the findings of the study, which highlighted the above-mentioned pathways enriched by FAP and its co-expressed genes. A schematic overview of the functional role of FAP in driving tumorigenesis is depicted in Fig. 9. The clinical impact of the FAP across tumors was signified from the high HR and low median survival of the high expression FAP group. The evident decline in the median overall survival was witnessed in BLCA, BRCA, ESCA, KIRC, KIRP, LIHC and STAD, where the difference in the overall median survival was over 35 months [51].

The secondary structure of the FAP depicted a well-characterized α-helix, extended strand, β-turns and random coil regions in the protein. The well-characterized structure of FAP consisting of a seven-bladed β propeller domain on top of the catalytic triad serves as a selective filter to reach the catalytic domain [52]. The negative Z-score value estimated from ProSA-web energetics proposed a good and stable protein model with nominal misfolds and erroneous conformation. Further, evidence for the stability of the protein was indicated by the instability index [53]. The catalytic triad of FAP consisting of serine, aspartate and histidine at 624, 702 and 734 amino acid positions respectively, was validated from the InterPro domain (IPR001375). The domain is responsible for the endopeptidase and dipeptidyl peptidase activity of FAP [7]. The cavity formed by the catalytic triad residues offers more access for substrate binding and is also located at the interface of the highly characterized β propeller domain giving substrate specificity to the cavity [54]. MDS studies computed a low RMSD value depicting minimal variations in the backbone atoms of the protein across the course of the simulation. Thereafter, the RMSF profile of the protein identified the catalytic domain to be less fluctuating when compared to the overall protein. This suggests that the catalytic domain to be stable with less flexibility during the course of the simulation. The overall stability of the protein with minimal fluctuations is essential for maintaining the functional role of modulating the architectural and composition of the ECM, aiding in tumor progression and metastasis [55]. While the stability of the protein was evaluated through the R_g_ plot that depicted an increase in the compactness of the protein with time; the potential energy plot indicated an energetically favourable trajectory for the protein. The increasing compactness aids the FAP in binding to ECM components such as the degraded collagens and integrins for ECM remodeling [54]. The SASA area and free energy solvation plot further complemented the stability and folding of the protein in an aqueous environment [56, 57]. The stable hydrophobic compactness of the protein is crucial for determining the stability as well as binding of the inhibitor to the active site [58].

The mutational profiling of the FAP across various tumors identified five deleterious and stabilizing mutations (with decreasing flexibility) to be frequently mutated (≥ 2 mutations) in the catalytic domain [59]. This was significant in determining as mutants in the catalytic domain can hinder the binding efficiency of lead molecules designed to target the protein. For the present study, we also considered the catalytically inactive form of FAP (FAP_S624A), as inactive FAP was also reported to be activating the PI3K pathway for tumor proliferation [60]. All the mutants generated were studied for the backbone dynamics evaluated using the N–H S^2^ order parameter. Mutations such as FAP_S576V, FAP_S624A, and FAP_G666C were identified to decrease the backbone flexibility (> 0.8) when acquiring mutations, thereby further supporting the stability profile of the protein.

Mainly three standard drugs are administered against FAP, namely Val-boroPro (Talabostat), Glu-boroPro (PT-630) and LAF-237. Talabostat and PT-630 have been reported to decrease the stromal growth on FAP inhibition; however, the translational efficacy of the drugs was restricted due to the short half-life before resulting in the cyclization and loss of inhibitory activity [61]. This has led to the selection of LAF-237 as the standard for the ligand-based virtual screening. The analogs selected (based on similarity score) along with LAF-237 were subjected to PK profiling to establish high GI absorption, no Lipinski violations, no lead-like violations, low synthetic accessibility, no Pg substrate and no toxicity (LD_50_ > 1000 mg/kg) lead molecules. However, LAF-237 showed poor PK profile, which could be a probable reason for the undesirable survival outcomes in cancer patients [62]. The Lead 16 compound was identified to be interacting with the catalytic triad residues through these interactions, indicating its ability to hinder the catalytic property of FAP as well as the inactive FAP. Additionally, the endopeptidase activity of FAP is promoted by A657 and its conserved active sites consisting of R123, E203, E204, Y656 and N704 [63]. The intermolecular interactions of Lead 16 with these active sites residues confirms the endopeptidase inhibitory effect of Lead 16 (Online Resource 7). The docked complex of FAP targets with Lead 16 also showed decreased RMSF indicating a stable protein–ligand binding. Inhibiting the endopeptidase activity of FAP, results in the prevention of ECM remodeling, necessary for tumor growth. FAP involves alteration of ECM to promote tumor invasion by the integrin-FAK mediation [55]. Therefore, the inhibition of the FAP activity can impede tumor invasion. Also, endopeptidase activity of FAP leads to the ECM remodeling of TME resulting in an immunosuppressive environment. This is achieved by the ability of FAP to induce CCL2 secretion via the STAT/CCL2 signaling [64, 65]. Reports of FAP’s ability to induce EMT solely through the modulation of transcriptional factors such as Slug and Snail also suggest the important role of FAP in tumor metastasis [66]. Experimental evidence through immunohistochemistry techniques have shown the elevated expression of FAP in colorectal cancer. The expression of FAP was also associated with poor prognosis, advanced stages, and activation of angiogenesis and collagen degradation [67]. Further, the transition to malignant dysplasia in pancreatic ductal carcinoma was marked by changes in the expression pattern of FAP [68]. Tumor promotion induced by FAP via epithelial-mesenchymal transition (EMT) was observed in oral squamous cell carcinoma (OSCC). FAP down-regulated DPP9 in non-enzymatic way and led to promote EMT in OSCC [69]. Experimental reports on FAP’s role in tumorigenesis were further validated by Dong et al. [70], where Polyphyllin I inhibited gastric cancer cell growth by downregulating the expression of FAP and hepatocyte growth factor. Moreover, elevated expression of FAP associated with chemoresistance and poor prognosis was reported in gastric cancer [43]. Therefore, a potent lead compound that can efficiently inhibit the FAP activity can be of clinical advantage. The Lead 16, with better synthetic accessibility, non-toxic profile and stable intermolecular interactions with endopeptidase domain residues can be a better candidate for further experimental studies.

## Limitations

The present study utilizes a very detailed computational approach in elucidating the therapeutic potential of FAP for progressive tumors. Hence, experiments using in vitro and in vivo models are required to validate and confirm the insights of the present study. The study also relied on publicly available datasets; therefore, inclusion of external validation cohorts could sufficiently enhance the credibility of the studies.

## Conclusion

The serine protease FAP detected on the surface of CAFs is tumor-specific in their expression pattern and has low overall survival across various tumors. Additionally, the pro-tumorigenic functional role of FAP to remodel the cancer tissue promoting cell proliferation, migration and invasion can be targeted as part of therapeutic intervention. FEA identified biological pathways such as focal adhesion, PI3K/Akt signaling and proteoglycans in cancer aid in the migration of tumor cells. The present study identified FAP and its stabilizing mutants of the catalytic domain as well as inactive FAP to be targeted by pyrrolidine analogs. The identified lead molecule exhibited stable intermolecular interactions with crucial amino acid residues for endopeptidase activity. The ability of the lead molecule to form intermolecular interactions with the catalytic triad amino acid residues encourages us to confirm the inhibitory role of the lead molecule against FAP. Experimental investigations through in vitro-in vivo models can further validate the inhibitory role of the pyrrolidine analog as cancer therapeutic.

## Supplementary Information

Supplementary material 1.

Supplementary material 2.

Supplementary material 3.

Supplementary material 4.

Supplementary material 5.

Supplementary material 6.

Supplementary material 7.

Supplementary material 8.

## Data Availability

The data that supports the findings of this study is provided in the Supplementary Files.
