# Supplementary material for: Transcriptomic, mutational and structural bioinformatics approaches to explore the therapeutic role of FAP in predominant cancer types
Source: Discov Oncol. 2024 Nov 23;15:699. doi: 10.1007/s12672-024-01531-x (PMC11585531; doi:10.1007/s12672-024-01531-x)
Supplement: Supplementary file 1 — Supplementary material 1. [file 12672_2024_1531_MOESM1_ESM.pdf]

Transcriptomic, mutational and structural bioinformatics approaches to explore the therapeutic role of FAP in predominant cancer types

Gayathri Ashok[1,2], Abdullah F AlAsmari[3], Fawaz AlAsmari[3], Paul Livingstone[4], Anand Anbarasu[1,5], Sudha Ramaiah[1,2]*

[1]*Medical and Biological Computing Laboratory, School of Biosciences and Technology (SBST), Vellore Institute of Technology (VIT), Vellore-632014, Tamil Nadu, India*

[2]*Department of Bio-Sciences, SBST, VIT, Vellore-632014, Tamil Nadu, India*

[3]*Department of Pharmacology and Toxicology, College of Pharmacy, King Saud University,13 Riyadh 11451, Saudi Arabia*

[4]*School of Sports and Health Sciences, Cardiff Metropolitan University, Cardiff CF5 2YB, UK*

[5]*Department of Biotechnology, SBST, VIT, Vellore-632014, Tamil Nadu, India*

***Corresponding author**

Prof. (Dr.) Sudha Ramaiah

Medical and Biological Computing Laboratory

School of Biosciences and Technology

VIT, Vellore-632014

Tamil Nadu, India

Tel: +91-416-2556/2694; Fax: +91-416-2243092

Email id: sudhaanand@vit.ac.in

Online Resource 1 Comparative gene expression profiling of FAP across TCGA tumor OncoDB

Cancer	Log2FC	P-value
ACC	0.26	3.4e-04
BLCA	0.78	2.0e-10
BRCA	1.52	1.9e -72
CESC	-2.26	1.8e -03
CHOL	2.66	4.5e-06
COAD	3.22	2.4e-23
ESCA	3.05	2.7e-04
GBM	1.58	6.9e-07
HNSCC	3.72	4.6e-36
KICH	0.00	8.9e-01
KIRC	1.83	2.9e-03
KIRP	0.00	1.4e-01
LGG	0.14	1.8e-13
LIHC	0.00	6.3e-16
LUAD	2.05	1.8e-47
LUSC	1.56	4.0e-25
OV	0.68	5.6e-15
PAAD	5.08	4.2e-34
PCPG	0.49	6.1e-04
PRAD	0.75	1.8e-08
READ	2.18	1.6e-05
SKCM	-0.12	3.9e-10
STAD	3.47	3.3e-47
TGCT	0.33	8.7e-07
THCA	0.18	7.6e-14
UCEC	-2.66	5.8e-05
UCS	-2.46	2.5e-25
