# Supplementary material for: Transcriptomic, mutational and structural bioinformatics approaches to explore the therapeutic role of FAP in predominant cancer types
Source: Discov Oncol. 2024 Nov 23;15:699. doi: 10.1007/s12672-024-01531-x (PMC11585531; doi:10.1007/s12672-024-01531-x)
Supplement: Supplementary file 2 — Supplementary material 2. [file 12672_2024_1531_MOESM2_ESM.pdf]

# Transcriptomic, mutational and structural bioinformatics approaches to explore the therapeutic role of FAP in predominant cancer types

**Gayathri Ashok[1,2], Abdullah F AlAsmari[3], Fawaz AlAsmari[3], Paul Livingstone[4], Anand Anbarasu[1,5], Sudha Ramaiah[1,2]***

[1]*Medical and Biological Computing Laboratory, School of Biosciences and Technology (SBST), Vellore Institute of Technology (VIT), Vellore-632014, Tamil Nadu, India*

[2]*Department of Bio-Sciences, SBST, VIT, Vellore-632014, Tamil Nadu, India*

[3]*Department of Pharmacology and Toxicology, College of Pharmacy, King Saud University,13 Riyadh 11451, Saudi Arabia*

[4]*School of Sports and Health Sciences, Cardiff Metropolitan University, Cardiff CF5 2YB, UK*

[5]*Department of Biotechnology, SBST, VIT, Vellore-632014, Tamil Nadu, India*

***Corresponding author**

Prof. (Dr.) Sudha Ramaiah

Medical and Biological Computing Laboratory

School of Biosciences and Technology

VIT, Vellore-632014

Tamil Nadu, India

Tel: +91-416-2556/2694; Fax: +91-416-2243092

Email id: sudhaanand@vit.ac.in

**Online Resource 2** Co-expression proteins with Pearson correlation coefficient using GEPIA [PCC ≥ 0.5]

| Gene name | ENSEMBL ID | Pearson Corelation Coefficient (PCC) |
|---|---|---|
| *COL5A2* | ENSG00000204262.11 | 0.76 |
| *COL6A3* | ENSG00000163359.15 | 0.73 |
| *MMP2* | ENSG00000087245.12 | 0.72 |
| *COL1A2* | ENSG00000164692.17 | 0.72 |
| *CTHRC1* | ENSG00000164932.12 | 0.72 |
| *COL3A1* | ENSG00000168542.12 | 0.7 |
| *COL5A1* | ENSG00000130635.15 | 0.7 |
| *ADAM12* | ENSG00000148848.14 | 0.7 |
| *COL12A1* | ENSG00000111799.20 | 0.69 |
| *ADAMTS12* | ENSG00000151388.10 | 0.68 |
| *COL1A1* | ENSG00000108821.13 | 0.67 |
| *POSTN* | ENSG00000133110.14 | 0.67 |
| *LRRC15* | ENSG00000172061.8 | 0.65 |
| *MXRA5* | ENSG00000101825.7 | 0.64 |
| *LUM* | ENSG00000139329.4 | 0.63 |
| *ANTXR1* | ENSG00000169604.19 | 0.61 |
| *THBS2* | ENSG00000186340.14 | 0.61 |
| *AC093850.2* | ENSG00000230838.1 | 0.61 |
| *ADAMTS2* | ENSG00000087116.13 | 0.61 |
| *PDGFRB* | ENSG00000113721.13 | 0.6 |
| *ITGA11* | ENSG00000137809.16 | 0.59 |
| *MMP14* | ENSG00000157227.12 | 0.59 |
| *ASPN* | ENSG00000106819.11 | 0.59 |
| *COL11A1* | ENSG00000060718.18 | 0.58 |
| *COL6A2* | ENSG00000142173.14 | 0.57 |
| *COL6A1* | ENSG00000142156.14 | 0.57 |
| *SFRP2* | ENSG00000145423.4 | 0.57 |
| *BGN* | ENSG00000182492.15 | 0.57 |
| *CERCAM* | ENSG00000167123.18 | 0.56 |
| *GLT8D2* | ENSG00000120820.12 | 0.56 |
| *COL10A1* | ENSG00000123500.9 | 0.56 |
| *RP11-417E7.2* | ENSG00000261039.2 | 0.56 |
| *ITGBL1* | ENSG00000198542.13 | 0.56 |
| *AEBP1* | ENSG00000106624.8 | 0.56 |
| *SULF1* | ENSG00000137573.13 | 0.55 |
| *CDH11* | ENSG00000140937.13 | 0.54 |
| *SPARC* | ENSG00000113140.10 | 0.54 |
| *RP11-863P13.3* | ENSG00000261327.4 | 0.54 |
| *MMP11* | ENSG00000099953.9 | 0.54 |
| *RCN3* | ENSG00000142552.7 | 0.53 |
| *GPX8* | ENSG00000164294.13 | 0.52 |
| *ITGB5* | ENSG00000082781.11 | 0.52 |

| | | |
|---|---|---|
| *C1QTNF6* | ENSG00000133466.13 | 0.52 |
| *FBN1* | ENSG00000166147.13 | 0.52 |
| *TMEM119* | ENSG00000183160.8 | 0.52 |
| *LOXL1* | ENSG00000129038.15 | 0.52 |
| *WISP1* | ENSG00000104415.13 | 0.52 |
| *CMTM3* | ENSG00000140931.19 | 0.51 |
| *SERPINH1* | ENSG00000149257.13 | 0.51 |
| *P4HA3* | ENSG00000149380.11 | 0.51 |
| *PODN* | ENSG00000174348.13 | 0.51 |
| *CRISPLD2* | ENSG00000103196.11 | 0.51 |
| *OLFML2B* | ENSG00000162745.10 | 0.51 |
| *MRC2* | ENSG00000011028.13 | 0.51 |
| *SH3PXD2B* | ENSG00000174705.11 | 0.51 |
| *PRRX1* | ENSG00000116132.11 | 0.51 |
| *DCN* | ENSG00000011465.16 | 0.5 |
| *GXYLT2* | ENSG00000172986.12 | 0.5 |
| *RP3-495K2.2* | ENSG00000229720.1 | 0.5 |
