# Supplementary material for: Transcriptomic, mutational and structural bioinformatics approaches to explore the therapeutic role of FAP in predominant cancer types
Source: Discov Oncol. 2024 Nov 23;15:699. doi: 10.1007/s12672-024-01531-x (PMC11585531; doi:10.1007/s12672-024-01531-x)
Supplement: Supplementary file 3 — Supplementary material 3. [file 12672_2024_1531_MOESM3_ESM.pdf]

Transcriptomic, mutational and structural bioinformatics approaches to explore the therapeutic role of FAP in predominant cancer types

Gayathri Ashok[1,2], Abdullah F AlAsmari[3], Fawaz AlAsmari[3], Paul Livingstone[4], Anand Anbarasu[1,5], Sudha Ramaiah[1,2]*

[1]*Medical and Biological Computing Laboratory, School of Biosciences and Technology (SBST), Vellore Institute of Technology (VIT), Vellore-632014, Tamil Nadu, India*

[2]*Department of Bio-Sciences, SBST, VIT, Vellore-632014, Tamil Nadu, India*

[3]*Department of Pharmacology and Toxicology, College of Pharmacy, King Saud University,13 Riyadh 11451, Saudi Arabia*

[4]*School of Sports and Health Sciences, Cardiff Metropolitan University, Cardiff CF5 2YB, UK*

[5]*Department of Biotechnology, SBST, VIT, Vellore-632014, Tamil Nadu, India*

***Corresponding author**

Prof. (Dr.) Sudha Ramaiah

Medical and Biological Computing Laboratory

School of Biosciences and Technology

VIT, Vellore-632014

Tamil Nadu, India

Tel: +91-416-2556/2694; Fax: +91-416-2243092

Email id: sudhaanand@vit.ac.in

Online Resource 3 Pan-cancer survival analysis using Kmplotter

Cancer	HR	p-value	High expression cohort	Low expression cohort
BLCA	1.58	0.008	28.63	106.1
BRCA	1.69	0.011	116.4	248.5
CESC	1.81	0.011	25.77	48.43
ESCA	1.81	0.136	24.33	59.37
HNSCC	1.61	0.0005	33.27	58.73
KIRC	1.62	0.001	74.23	118.47
KIRP	3.82	2.1e-06	21.93	86.97
LIHC	1.56	0.02	47.43	82.87
LUAD	1.36	0.05	45.97	55.1
LUSC	1.42	0.01	35.83	61.37
OV	1.27	0.07	41.97	48.2
PDAC	1.5	0.07	19.77	35.3
READ	0.59	0.18	47.73	36.53
SARC	0.82	0.38	28.33	29.9
STAD	1.69	0.001	20.23	56.2
