# Supplementary material for: Transcriptomic, mutational and structural bioinformatics approaches to explore the therapeutic role of FAP in predominant cancer types
Source: Discov Oncol. 2024 Nov 23;15:699. doi: 10.1007/s12672-024-01531-x (PMC11585531; doi:10.1007/s12672-024-01531-x)
Supplement: Supplementary file 5 — Supplementary material 5. [file 12672_2024_1531_MOESM5_ESM.pdf]

Transcriptomic, mutational and structural bioinformatics approaches to explore the therapeutic role of FAP in predominant cancer types

Gayathri Ashok[1,2], Abdullah F AlAsmari[3], Fawaz AlAsmari[3], Paul Livingstone[4], Anand Anbarasu[1,5], Sudha Ramaiah[1,2]*

[1]*Medical and Biological Computing Laboratory, School of Biosciences and Technology (SBST), Vellore Institute of Technology (VIT), Vellore-632014, Tamil Nadu, India*

[2]*Department of Bio-Sciences, SBST, VIT, Vellore-632014, Tamil Nadu, India*

[3]*Department of Pharmacology and Toxicology, College of Pharmacy, King Saud University,13 Riyadh 11451, Saudi Arabia*

[4]*School of Sports and Health Sciences, Cardiff Metropolitan University, Cardiff CF5 2YB, UK*

[5]*Department of Biotechnology, SBST, VIT, Vellore-632014, Tamil Nadu, India*

***Corresponding author**

Prof. (Dr.) Sudha Ramaiah

Medical and Biological Computing Laboratory

School of Biosciences and Technology

VIT, Vellore-632014

Tamil Nadu, India

Tel: +91-416-2556/2694; Fax: +91-416-2243092

Email id: sudhaanand@vit.ac.in

Online Resource 5A Pan-cancer mutational profiling of FAP

Ref Residue	Protein Position	Alt Residue	Cancer Type	Frequency	Frequency in overall cancer
D	360	N	Adrenal gland cancer	2	2
M	39	I	Breast cancer	1	5
L	90	F		1	
L	114	P		1	
T	404	A		1	
S	407	L		1	
D	406	N	Cervical cancer	1	2
I	501	V		1	
A	41	T	Colorectal cancer	1	26
P	149	T		1	
D	198	E		1	
F	220	C		2	
R	263	W		1	
V	282	I		1	
T	297	M		2	
F	323	C		2	
F	350	L		1	
S	363	L		1	
S	407	L		1	
Q	502	H		2	
Y	541	C		1	
T	577	I		1	
T	602	A		2	
R	605	G		3	
I	620	M		2	
T	681	N		1	
S	237	P	Esophageal cancer	1	9
Y	239	C		1	
A	273	V		1	
V	315	A		1	
S	363	L		1	
Y	451	C		1	
Y	625	C		1	
V	629	I		1	
N	742	Y		1	
R	303	L	Head and neck cancer	1	9
N	413	S		1	
D	457	N		2	
E	609	A		2	
S	710	L		1	
T	741	K		2	

E	512	D	Hematologic cancer	1	3
N	556	Y		1	
L	584	H		1	
L	68	P	Kidney cancer	1	2
E	495	K		1	
L	78	I	Liver cancer	1	20
T	83	K		1	
P	107	H		1	
Q	110	K		1	
L	121	I		1	
T	269	N		1	
T	300	N		1	
S	319	Y		1	
S	358	R		1	
A	361	D		1	
Q	405	K		1	
N	423	K		1	
S	428	R		1	
R	480	S		1	
P	526	H		1	
A	554	D		1	
G	576	V		2	
A	619	D		1	
G	637	E		1	
R	27	C	Lung cancer	2	40
T	51	R		1	
K	55	T		2	
M	95	V		5	
Y	113	F		1	
P	179	Q		1	
P	216	T		1	
I	231	M		2	
Q	307	R		1	
R	324	K		1	
E	325	K		1	
K	334	N		1	
G	349	V		2	
G	373	C		1	
E	396	G		2	
V	439	I		1	
R	444	M		1	
E	489	D		1	
L	511	I		1	
R	590	Q		4	
A	633	S		1	
A	648	V		1	

G	666	C		2	
T	700	I		1	
S	737	Y		1	
G	738	C		2	
S	435	I	Malignant glioma	1	14
N	556	Y		8	
G	581	S		2	
L	674	V		1	
E	675	D		1	
A	711	S		1	
P	59	S	Melanoma	1	60
G	64	A		1	
P	149	S		3	
P	179	S		3	
G	195	R		3	
E	204	K		3	
D	241	N		1	
E	242	K		1	
N	259	K		1	
P	272	S		2	
S	363	L		3	
H	376	R		3	
E	385	K		7	
R	421	K		1	
F	455	I		1	
P	472	S		3	
G	479	E		3	
S	531	L		2	
P	535	S		3	
R	590	Q		3	
S	631	L		1	
G	637	E		4	
G	699	E		2	
S	757	F		5	
P	245	L	Ovarian cancer	2	4
D	301	N		1	
T	741	K		1	
S	71	F	Pancreatic cancer	3	11
Y	124	F		1	
R	142	I		1	
R	148	H		1	
I	183	M		1	
E	302	K		1	
R	303	G		1	
H	477	P		1	
V	588	M		1	

N	227	Y	Sarcoma	1	2
R	311	G		1	
S	369	R	Skin cancer	1	1
S	316	L	Stomach cancer	4	4
C	23	Y	Urinary bladder cancer	1	5
Y	79	F		1	
P	179	S		1	
S	342	R		1	
Q	539	E		1	
W	4	S	Uterine cancer	2	30
A	72	T		1	
V	77	I		1	
L	162	S		2	
A	163	T		1	
K	219	T		1	
D	241	N		1	
R	263	Q		1	
S	316	L		1	
K	334	N		2	
D	382	Y		1	
A	387	D		1	
D	406	E		1	
S	407	L		1	
F	415	S		1	
I	429	T		1	
S	474	F		1	
E	484	V		1	
N	491	I		1	
N	500	D		1	
R	530	I		1	
R	590	Q		1	
G	593	D		1	
A	603	T		1	
A	657	V		1	
Y	660	C		1	
F	664	L		1	

Online Resource 5B Stabilizing and deleterious mutations with a frequency ≥ 2 in FAP

**Ref Residue**	**Protein Position**	**Alt Residue**	**Cancer Type**	**Frequency**	**DDS (kcal.mol-¹.K-¹)**	**Flexibility**	**DDG (kcal/mol)**	**Stability**	**Functional Impact**
S	71	F	Pancreatic Cancer	3	-0.023	Decrease	0.323	Stabilizing	Deleterious
E	204	K	Melanoma	3	-0.197	Decrease	0.622	Stabilizing	Deleterious
I	231	M	Lung Cancer	2	-0.176	Decrease	0.328	Stabilizing	Deleterious
P	245	L	Ovarian Cancer	2	-0.422	Decrease	0.413	Stabilizing	Deleterious
R	263	W	Colorectal Cancer	1	-0.771	Decrease	1.408	Stabilizing	Deleterious
T	297	M	Colorectal Cancer	2	-0.332	Decrease	0.788	Stabilizing	Deleterious
H	376	R	Melanoma	3	-0.082	Decrease	0.163	Stabilizing	Deleterious
G	479	E	Melanoma	3	-0.742	Decrease	0.477	Stabilizing	Deleterious
S	531	L	Melanoma	2	-0.014	Decrease	0.072	Stabilizing	Deleterious
P	535	S	Melanoma	3	0.094	Increase	0.131	Stabilizing	Deleterious
**G**	**576**	**V**	**Liver Cancer**	**2**	**-0.973**	**Decrease**	**0.045**	**Stabilizing**	**Deleterious**
**G**	**581**	**S**	**Malignant Glioma**	**2**	**-0.486**	**Decrease**	**0.038**	**Stabilizing**	**Deleterious**
**I**	**620**	**M**	**Colorectal Cancer**	**2**	**-0.492**	**Decrease**	**0.009**	**Stabilizing**	**Deleterious**
**G**	**666**	**C**	**Lung Cancer**	**2**	**-0.543**	**Decrease**	**0.147**	**Stabilizing**	**Deleterious**
**S**	**757**	**F**	**Melanoma**	**5**	**-0.166**	**Decrease**	**1.289**	**Stabilizing**	**Deleterious**

*Bold indicates the mutations present in the catalytic domain
