# Supplementary material for: Transcriptomic, mutational and structural bioinformatics approaches to explore the therapeutic role of FAP in predominant cancer types
Source: Discov Oncol. 2024 Nov 23;15:699. doi: 10.1007/s12672-024-01531-x (PMC11585531; doi:10.1007/s12672-024-01531-x)
Supplement: Supplementary file 6 — Supplementary material 6. [file 12672_2024_1531_MOESM6_ESM.pdf]

Transcriptomic, mutational and structural bioinformatics approaches to explore the therapeutic role of FAP in predominant cancer types

Gayathri Ashok[1,2], Abdullah F AlAsmari[3], Fawaz AlAsmari[3], Paul Livingstone[4], Anand Anbarasu[1,5], Sudha Ramaiah[1,2]*

[1]*Medical and Biological Computing Laboratory, School of Biosciences and Technology (SBST), Vellore Institute of Technology (VIT), Vellore-632014, Tamil Nadu, India*

[2]*Department of Bio-Sciences, SBST, VIT, Vellore-632014, Tamil Nadu, India*

[3]*Department of Pharmacology and Toxicology, College of Pharmacy, King Saud University,13 Riyadh 11451, Saudi Arabia*

[4]*School of Sports and Health Sciences, Cardiff Metropolitan University, Cardiff CF5 2YB, UK*

[5]*Department of Biotechnology, SBST, VIT, Vellore-632014, Tamil Nadu, India*

***Corresponding author**

Prof. (Dr.) Sudha Ramaiah

Medical and Biological Computing Laboratory

School of Biosciences and Technology

VIT, Vellore-632014

Tamil Nadu, India

Tel: +91-416-2556/2694; Fax: +91-416-2243092

Email id: sudhaanand@vit.ac.in

Online Resource 6 Drug-protein interaction network constructed using STITCH database

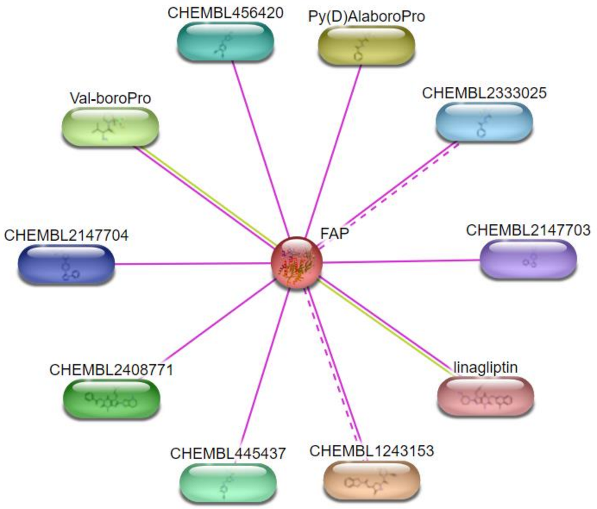

Node	Node 2	Drug ID	Experimentally determined interaction	Prediction	Combined score
FAP	CHEMBL1243153	CID046917048	0.898	0.401	0.937
	Py(D)AlaboroPro	CID071655266	0.867	0	0.867
	Val-boroPro	CID006918572	0.827	0	0.842
	CHEMBL2408771	CID066954220	0.838	0	0.838
	CHEMBL445437	CID044590651	0.832	0	0.832
	CHEMBL2333025	CID071655265	0.812	0.105	0.827
	CHEMBL456420	CID044590650	0.814	0	0.814
	CHEMBL2147704	CID067507176	0.81	0	0.81
	CHEMBL2147703	CID067507912	0.807	0	0.807
	Linagliptin	CID010096344	0.708	0	0.804
