# Supplementary material for: Transcriptomic, mutational and structural bioinformatics approaches to explore the therapeutic role of FAP in predominant cancer types
Source: Discov Oncol. 2024 Nov 23;15:699. doi: 10.1007/s12672-024-01531-x (PMC11585531; doi:10.1007/s12672-024-01531-x)
Supplement: Supplementary file 7 — Supplementary material 7. [file 12672_2024_1531_MOESM7_ESM.pdf]

Pan-cancer study exploring the therapeutic role of FAP by transcriptomic, mutational and structural bioinformatics approaches

Gayathri Ashok[1,2], Anand Anbarasu[1,3], Sudha Ramaiah[1,2]*

[1]*Medical and Biological Computing Laboratory, School of Biosciences and Technology (SBST), Vellore Institute of Technology (VIT), Vellore-632014, Tamil Nadu, India*

[2]*Department of Bio-Sciences, SBST, VIT, Vellore-632014, Tamil Nadu, India*

[3]*Department of Biotechnology, SBST, VIT, Vellore-632014, Tamil Nadu, India*

***Corresponding author**
Prof. (Dr.) Sudha Ramaiah
Medical and Biological Computing Laboratory
School of Biosciences and Technology
VIT, Vellore-632014
Tamil Nadu, India
Tel: +91-416-2556/2694; Fax: +91-416-2243092
Email id: sudhaanand@vit.ac.in

Author details

Sudha Ramaiah: Email: sudhaanand@vit.ac.in ORCID: https://orcid.org/0000-0002-4800-329X

Anand Anbarasu: Email: aanand@vit.ac.in ORCID**:** https://orcid.org/0000-0003-2216-7488

Gayathri Ashok: Email: gayathri.ashok@vit.ac.in ORCID: https://orcid.org/0000-0001-5018-4125

Acknowledgement

The authors would like to thank VIT management for providing the necessary facilities to carry out this research work. Ms. Gayathri Ashok would like to wholeheartedly thank Dr. Aniket Naha and Dr. Soumya Basu for their intellectual input and support for the manuscript.

Supplementary File 7A Binding affinity profile of lead compounds compared with LAF-237 across all protein targets

**Lead Molecules**	**FAP**		**FAP_S576V**		**FAP_G581S**		**FAP_I620M**		**FAP_S624A**		**FAP_G666C**		**Average BE (kcal /mol)**	**Averag e IC (µM)**
	**BE (kcal/ mol)**	**IC (µM)**	**BE (kcal /mol)**	**IC (µM)**	**BE (kcal /mol)**	**IC (µM)**	**BE (kcal /mol)**	**IC (µM)**	**BE (kcal /mol)**	**IC (µM)**	**BE (kcal /mol)**	**IC (µM)**		
Lead 16	**-6.07**	**35.25**	**-7.26**	**4.73**	**-6.76**	**11.06**	**-7.17**	**5.55**	**-7.31**	**4.39**	**-6.68**	**12.67**	**-6.87**	12.27
Lead 8	-6.71	12.13	-6.8	10.44	-7.01	7.31	-6.44	18.91	-6.39	20.59	-6.66	13.02	-6.66	13.73
Lead 21	-5.93	45.32	-6.14	31.75	-6.32	23.14	-6.36	21.8	-6.27	25.55	-6.56	15.58	-6.26	27.19
Lead 29	-6.36	21.62	-5.9	47.16	-6.01	39.06	-6.01	39.31	-5.95	43.16	-6.24	26.63	-6.07	36.15
Lead 17	-5.51	92.06	-6.01	39.25	-6.07	35.73	-6	39.7	-6.47	18.04	-6.21	28.25	-6.04	42.17
LAF-237	-7.13	5.9	-7.12	6.08	-7.01	7.3	-7.11	6.19	-7.19	5.39	-7	7.37	-7.09	6.37

Supplementary File 7B Intermolecular interaction profiles of screened molecules with FAP and mutants

Protein target	Lead molecules	Protein binding residues				
		**H-bond**	**van der Waals**	**Carbon-Hydrogen**	**Pi-alkyl & alkyl**	**Pi-Sigma**
**FAP**	LAF-237	**HIS734**, VAL540	GLN539, GLY622, GLY626, GLY735	**SER624**, TRP623	TRP621, TYR541, **HIS734**, TRP623	
	Lead16	GLN539, ASN556	TRP557, VAL540, GLY626, GLY622, **HIS734**	**SER624**, TYR745	TRP621, ILE558, TYR541	TRP623
	Lead 8	GLN539	LEU571, SER548, ASN556, TRP557, TYR745, VAL540, GLY622, TRP623		ILE558, TYR541	TRP621
	Lead 21	TRP623	ARG123, GLY735, SER548, GLY626, GLN539, VAL540, GLY622, **SER624**		TYR541, **HIS734**	TRP621
	Lead 29	**SER624**, **HIS734**, GLU203	TYR656, ASN704, ARG123, GLU204, TYR660		TRP623, PHE350	
	Lead 17	TYR541, **HIS734**, **SER624**, ARG123	GLY735, TYR660, PHE350, TYR656, GLU203, ASN704		TYR541, TRP623, **HIS734**	TRP623
**FAP_G576V**	LAF-237	TYR660, TRP623, TYR656	SER546, GLY543, TYR541, GLY626, **SER624**, PRO544, TYR625, PHE350, ASN704, GLU203, ARG123, GLU204			
	Lead16	TYR625, PRO544, **SER624**, **HIS734**, TYR660	CYS545, PHE350, ASN704, ARG123, GLY543	SER546, **SER624**	TYR656, VAL650, VAL705, TRP653	
	Lead 8	TYR625, TRP623, TYR660	**HIS734**, TYR541, GLY626, GLY543, PRO544, PHE350, ARG123	**SER624**	TYR656, VAL705, VAL650, TRP653	
	Lead 21	ARG123	PHE350, GLY543, TYR625, SER546, GLY626, TRP623,		TYR656, TYR660, VAL705, TYR541	**HIS734**

			**SER624**, ASN704, VAL650, PRO544			
	Lead 29	TYR660, TRP623	PHE350, PRO544, SER546, GLY543, TYR625, GLY626, ARG123, **SER624**, ASN704, VAL705, TRP653		TYR541, **HIS734**, VAL650	TYR656
	Lead 17	TYR625, **HIS734**, ARG123	TRP653, ASN704, TRP623, GLY626, GLY543, SER546, GLU204	**SER624**	PHE350, TYR660, VAL650, VAL705, TYR656, TYR541	
**FAP_G581S**	LAF-237	TRP623, TYR656, TYR660	GLY543, SER546, TYR541, GLY626, TYR625, **SER624**, ARG123, GLU203, GLU204		PHE350	
	Lead 16	PRO544, TYR656, TYR660	CYS545, TYR625, TYR541, **SER624**, ASN704, GLU204, GLU203, ARG123	SER546, TYR660	PHE350, **HIS734**, VAL705, VAL650, TYR656	
	Lead 8	TRP623, TYR625, TYR656, TYR660	GLY627, TYR541, PRO544, ARG123, PHE350, GLU204, GLY543	**SER624**	VAL705, **HIS734**, VAL650, TRP653	
	Lead 21	**HIS734**, ARG123, **SER624**, TYR660	ASN704, VAL650, PRO544, SER546, GLY543, TYR541, GLY626, TRP623, TYR625		VAL705, TYR656, PHE350	TYR660
	Lead 29	TYR656, GLU204, ARG123	ASN704, **HIS734**, PRO544, SER546, TRP623, GLY626, **SER624**, TYR625, PHE350, GLU203	GLY543	TYR660, TYR541	
	Lead 17	TYR625, **SER624**, **HIS734**, ARG123	GLY626, TRP623, VAL705, VAL650, TRP653, SER546, GLY543	**SER624**	TYR541, TYR656, TYR660	
**FAP_I620M**	LAF-237	TRP623, TYR660, TYR656	GLY626, **SER624**, TYR625, TYR541, SER546, GLY543, PRO544, PHE350, ARG123, ASN704, GLU203, GLU204			

	Lead 16	PRO544, TYR625, **HIS734**, **SER624**, TYR660	CYS545, ASN704, ARG123, GLY543	SER546	PHE350, TRP653, VAL650, VAL705	TYR656
	Lead 8	GLY543, SER546, ARG123	GLY542, TYR541, GLY626, TRP623, PRO544, TYR625, **SER624**, ASN704, **HIS734**, VAL705, VAL650		PHE350, TYR660, TYR656	
	Lead 21	**HIS734**, **SER624**, ARG123, TYR660	ASN704, VAL650, SER546, PRO544, GLY543, TYR541, GLY626, TRP623, TYR625		VAL705, TYR656, PHE350	
	Lead 29	SER546, TYR625, TRP623, ARG123	TYR541, GLY626, PHE350, **HIS734**, **SER624**, ASN704, GLY543	TYR660	VAL650, VAL705, TYR656	
	Lead 17	ARG123	**SER624**, GLY543, SER546, GLY626, TRP623, TYR625, GLU204	TYR656	PHE350, TYR660, **HIS734**, VAL650, VAL705, TYR541	
**FAP_S624A**	LAF-237	TYR656, TYR660, TRP623	SER546, GLY543, GLY626, ARG123, GLU203, GLU204		PHE350, TYR541, TYR625, **ALA624**	
	Lead16	TYR660, ARG123, TRP623	GLU203, GLU204, PHE350, TYR625, GLY543, SER546, TYR541		VAL650, **ALA624**, **HIS734**, VAL705	TYR656
	Lead 8	**HIS734**, TYR660, TYR625		ARG123, PRO544, TRP653, GLY543, SER546, GLY626, TRP623	**ALA624**, VAL705, VAL650, TYR656, TYR541	
	Lead 21	ARG123, **HIS734**, TYR660	TRP623, GLY626, TYR541, GLY543, SER546, PRO544, VAL650, ASN704, TYR625		**ALA624**, VAL705, TYR656, PHE350	

	Lead 29	TYR660, TRP623	PHE350, PRO544, GLY543, SER546, GLY626, ARG123, **HIS734**, ASN704, VAL705, TRP653		VAL650, TYR625, **ALA624**, TYR541	TYR656
	Lead 17	TYR660, GLY543	TRP653, TYR625, VAL705, **HIS734**, TYR541, GLY542, SER546		**ALA624**, TYR656, VAL650, PRO544	PHE350
**FAP_G666C**	LAF-237	TYR656, TYR660, TRP623	ARG123, GLU203, GLU204, GLY543, SER546, **SER624**		PHE350, TYR541, TYR625	
	Lead16	**HIS734**, ARG123, TRP623	SER546, GLY543, TYR625, **SER624**, GLY626, GLY627, TRP653	TYR656	TYR660, VAL650, VAL705, PHE350, TYR541	
	Lead 8	TYR660, GLY543, SER546, ARG123	VAL650, VAL705, ASN704, **SER624**, TYR625, TRP623, GLY626, TYR541, GLY542, PRO544		PHE350, TYR656, **HIS734**	
	Lead 21	TRP623, TYR625, **HIS734**, **SER624**	ASN704, GLY626, VAL705, VAL650, PHE350, SER546, PRO544, GLY543	TYR656	TYR541	TYR660
	Lead 29	GLU204, **HIS734**	PHE350, PRO554, TYR625, TYR660, TYR656, ASN704, ARG123, VAL705, **SER624**, GLY543. TYR541, SER546			
	Lead 17	ARG123, TYR625, **HIS734**	GLU203, GLU204, PHE350, SER546, GLY626, TRP653, TRP623, TYR660, ASN704, GLY543	**SER624**	VAL650, VAL705. TYR541, TYR656	

*Amino acid residues in bold indicate catalytic triad residues
