# Supplementary material for: Transcriptomic, mutational and structural bioinformatics approaches to explore the therapeutic role of FAP in predominant cancer types
Source: Discov Oncol. 2024 Nov 23;15:699. doi: 10.1007/s12672-024-01531-x (PMC11585531; doi:10.1007/s12672-024-01531-x)

Pan-cancer study exploring the therapeutic role of FAP by transcriptomic, mutational and structural bioinformatics approaches

Gayathri Ashok[1,2], Anand Anbarasu[1,3], Sudha Ramaiah[1,2]*

[1]*Medical and Biological Computing Laboratory, School of Biosciences and Technology (SBST), Vellore Institute of Technology (VIT), Vellore-632014, Tamil Nadu, India*

[2]*Department of Bio-Sciences, SBST, VIT, Vellore-632014, Tamil Nadu, India*

[3]*Department of Biotechnology, SBST, VIT, Vellore-632014, Tamil Nadu, India*

***Corresponding author**
Prof. (Dr.) Sudha Ramaiah
Medical and Biological Computing Laboratory
School of Biosciences and Technology
VIT, Vellore-632014
Tamil Nadu, India
Tel: +91-416-2556/2694; Fax: +91-416-2243092
Email id: sudhaanand@vit.ac.in

Author details

Sudha Ramaiah: Email: sudhaanand@vit.ac.in ORCID: https://orcid.org/0000-0002-4800-329X

Anand Anbarasu: Email: aanand@vit.ac.in ORCID**:** https://orcid.org/0000-0003-2216-7488

Gayathri Ashok: Email: gayathri.ashok@vit.ac.in ORCID: https://orcid.org/0000-0001-5018-4125

Acknowledgement

The authors would like to thank VIT management for providing the necessary facilities to carry out this research work. Ms. Gayathri Ashok would like to wholeheartedly thank Dr. Aniket Naha and Dr. Soumya Basu for their intellectual input and support for the manuscript.

Supplementary File 8A Intermolecular interactions of docked complexes of protein targets with LAF-237 (a) FAP (b) FAP_G576V (c) FAP_G581S (d) FAP_I620M (e) FAP_S624A (f) FAP_G666C

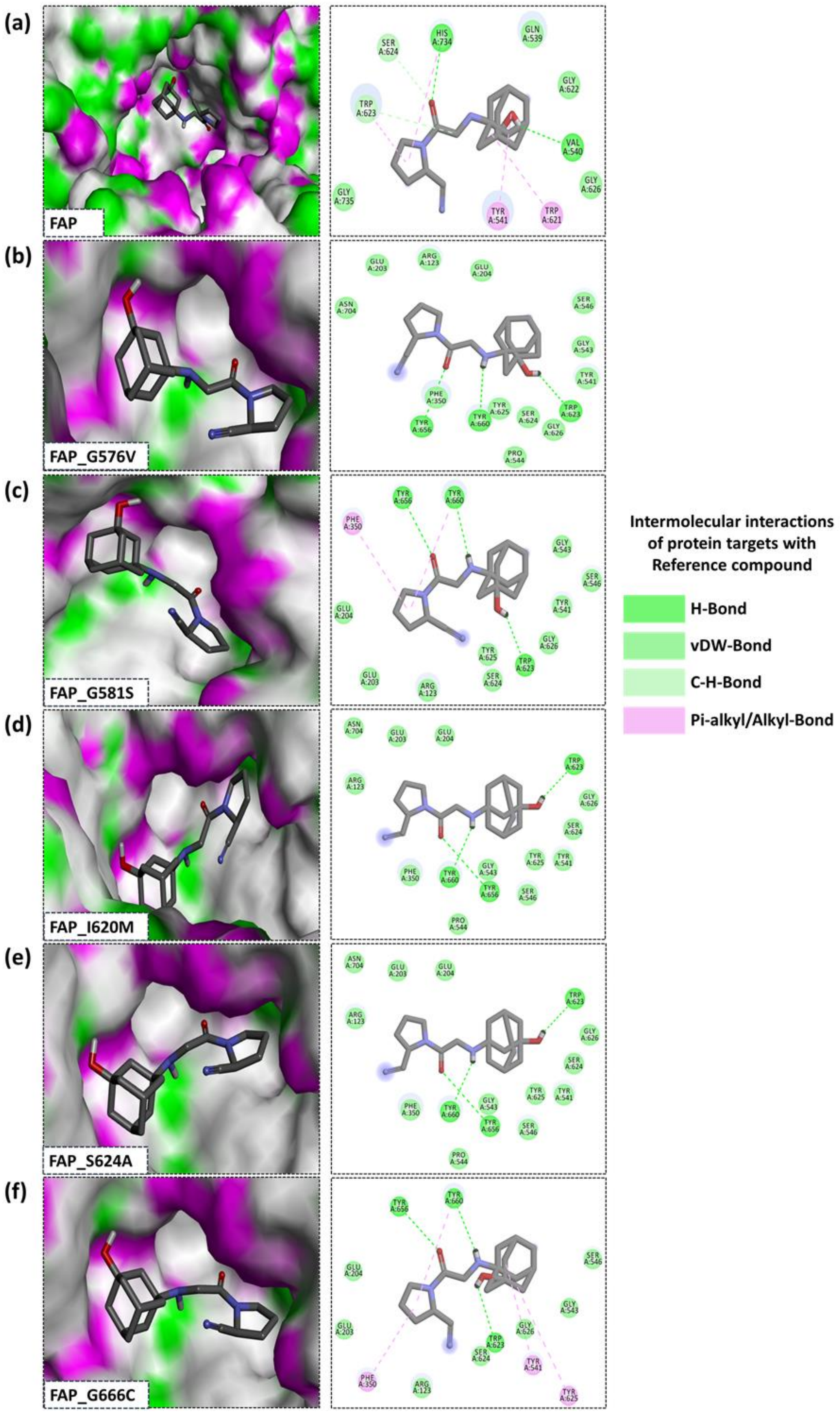

Supplementary File 8B Intermolecular interactions of docked complexes of protein targets with Lead 8 (a) FAP (b) FAP_G576V (c) FAP_G581S (d) FAP_I620M (e) FAP_S624A (f) FAP_G666C

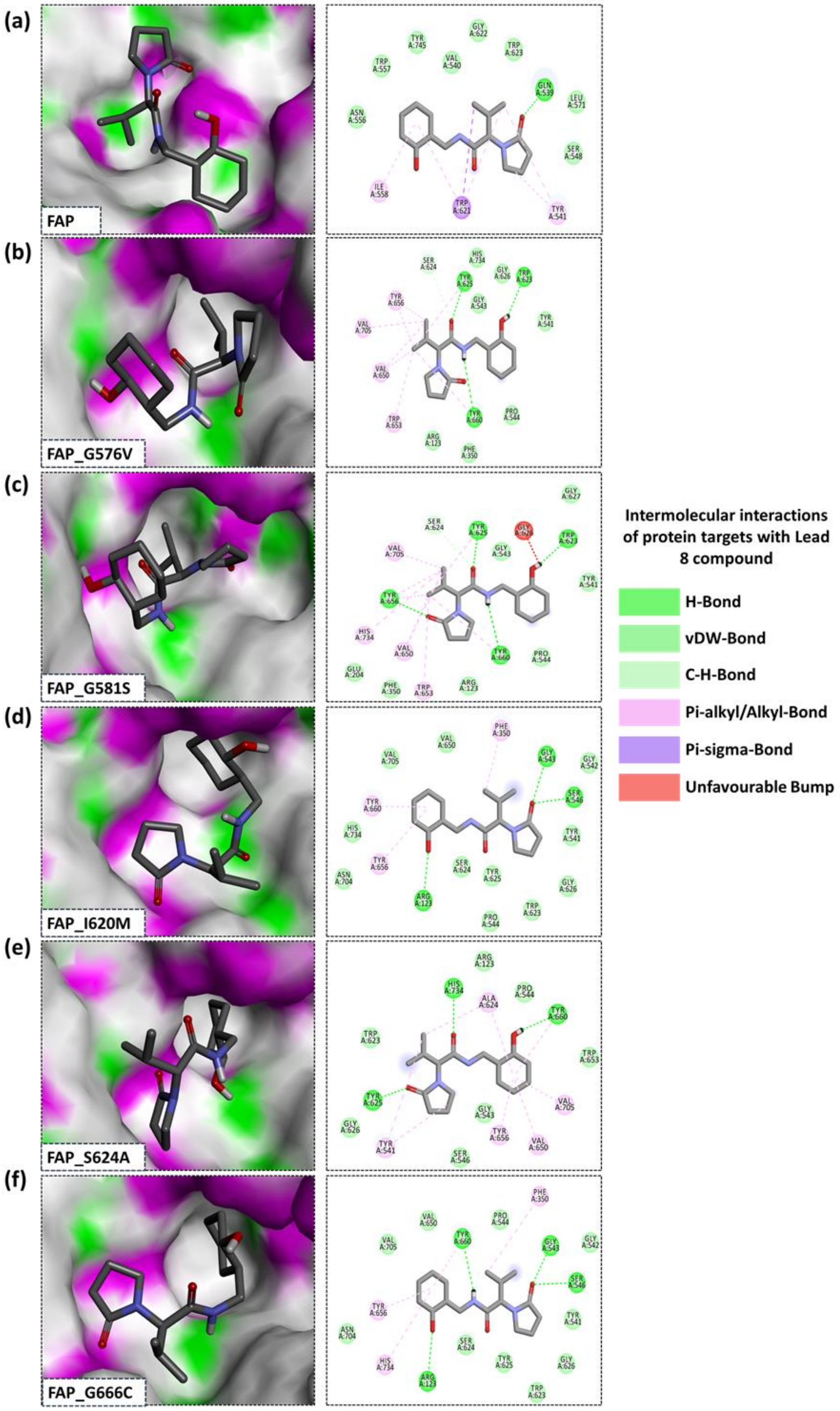

Supplementary File 8C Intermolecular interactions of docked complexes of protein targets with Lead 21 (a) FAP (b) FAP_G576V (c) FAP_G581S (d) FAP_I620M (e) FAP_S624A (f) FAP_G666C

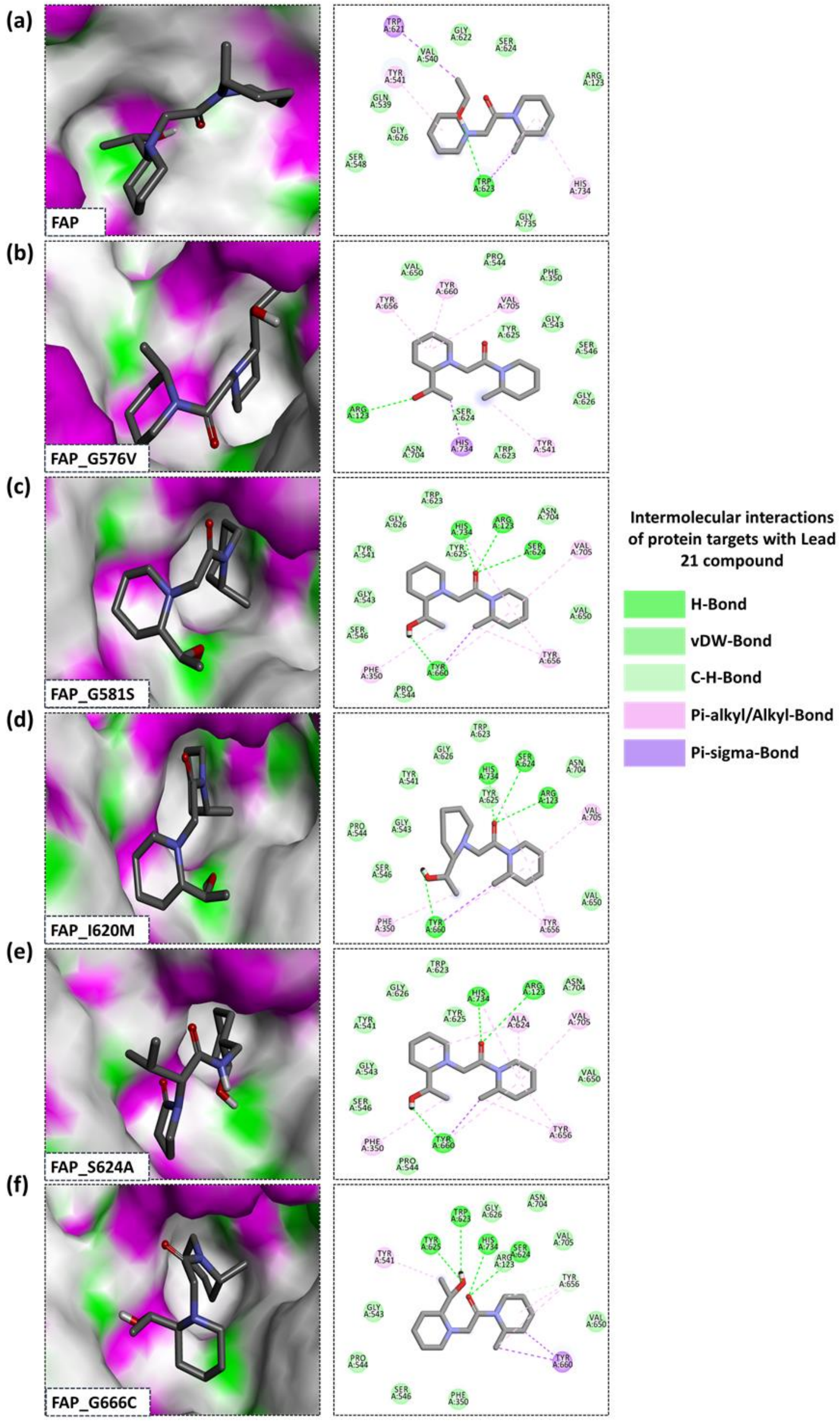

Supplementary File 8D Intermolecular interactions of docked complexes of protein targets with Lead 29 (a) FAP (b) FAP_G576V (c) FAP_G581S (d) FAP_I620M (e) FAP_S624A (f) FAP_G666C

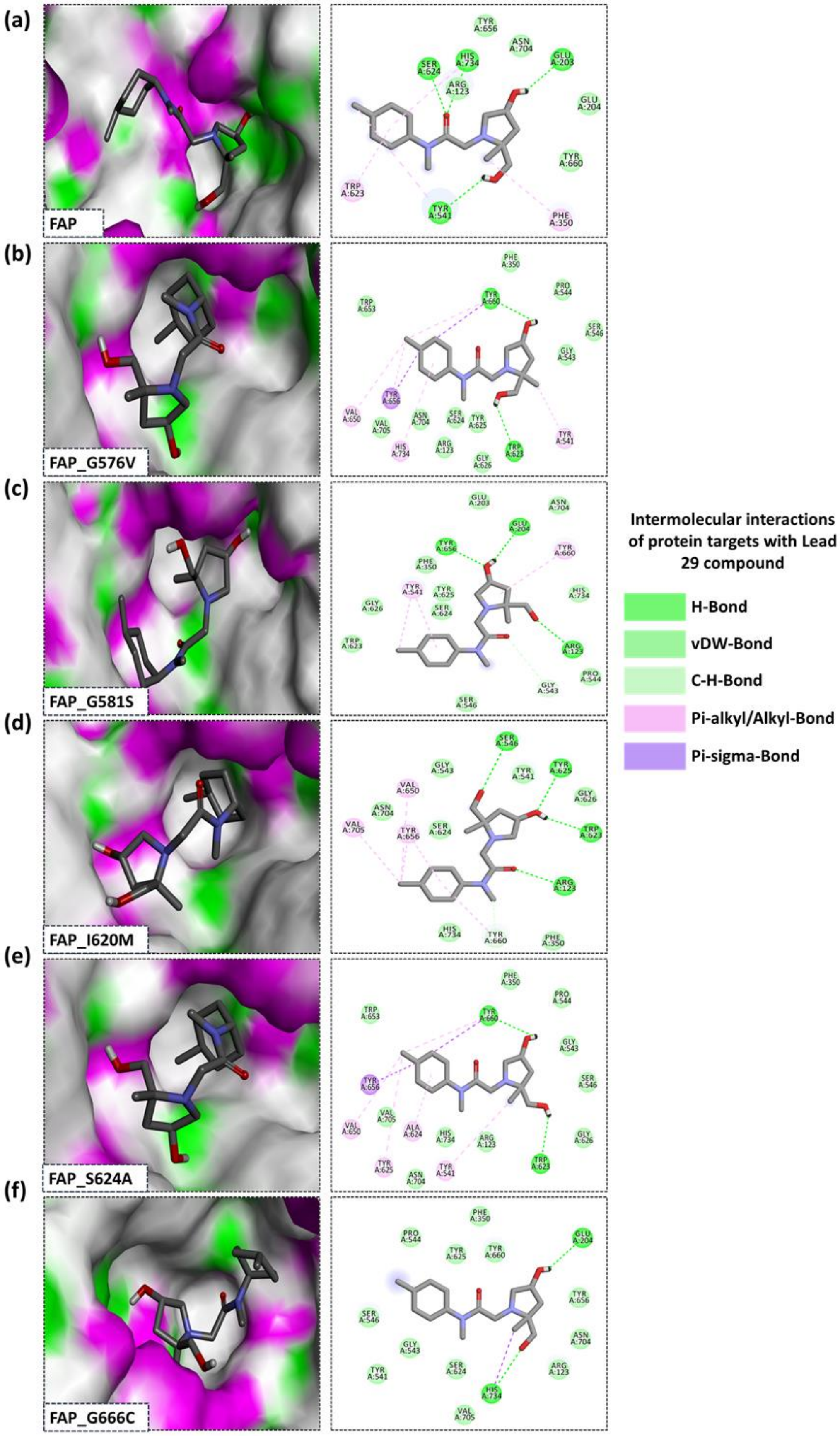

Supplementary File 8E Intermolecular interactions of docked complexes of protein targets with Lead 17 (a) FAP (b) FAP_G576V (c) FAP_G581S (d) FAP_I620M (e) FAP_S624A (f) FAP_G666C

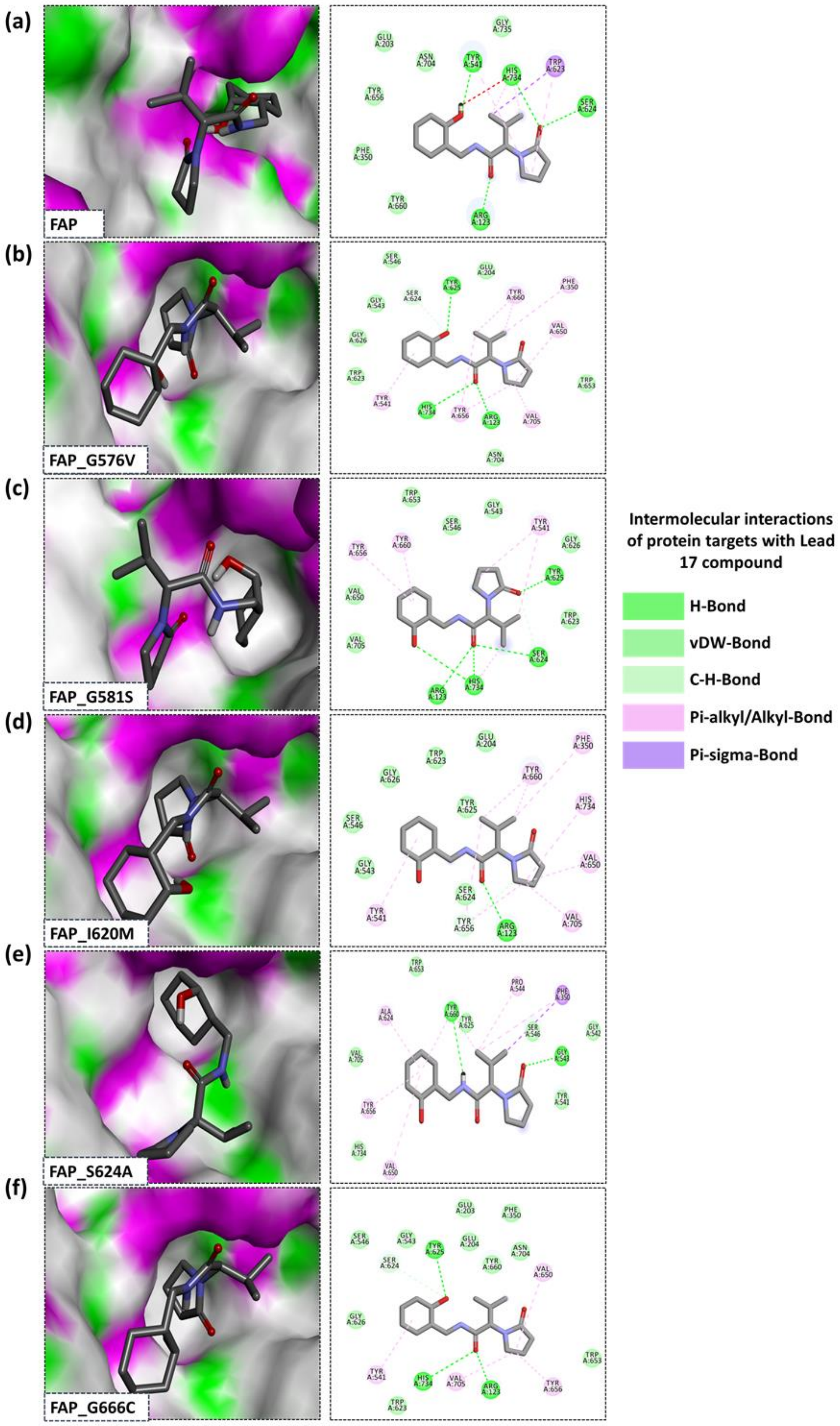

Supplement: Supplementary file 8 — Supplementary material 8. [file 12672_2024_1531_MOESM8_ESM.pdf]
